# On the Origin of *E*-Selectivity in the Ring-Opening Metathesis Polymerization with Molybdenum Imido Alkylidene *N-*Heterocyclic Carbene Complexes

**DOI:** 10.1021/acs.organomet.1c00229

**Published:** 2021-07-09

**Authors:** Maren Podewitz, Suman Sen, Michael R. Buchmeiser

**Affiliations:** † Institute of General, Inorganic and Theoretical Chemistry, and Center of Molecular Biosciences, University of Innsbruck, Innrain 80/82, AT-6020 Innsbruck, Austria; ‡ Institute of Polymer Chemistry, University of Stuttgart, Pfaffenwaldring 55, D-70569 Stuttgart, Germany

## Abstract

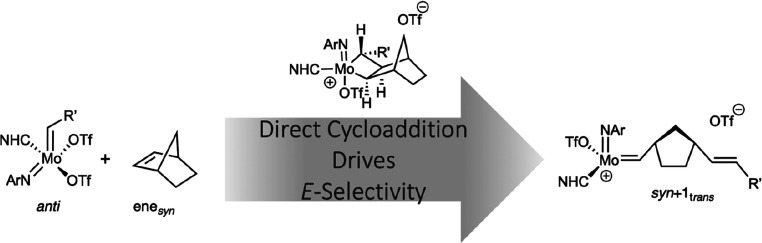

The understanding and control of stereoselectivity is a central aspect in ring-opening metathesis polymerization (ROMP). Herein, we report detailed quantum chemical studies on the reaction mechanism of *E*-selective ROMP of norborn-2-ene (NBE) with Mo(*N*-2,6-Me_2_-C_6_H_3_)(CHCMe_3_)(IMes)(OTf)_2_ (**1**, IMes = 1,3-dimesitylimidazol-2-ylidene) as a first step to stereoselective polymerization. Four different reaction pathways based on an ene_*syn*_ or ene_*anti*_ approach of NBE to either the *syn*- or *anti*-isomer of the neutral precatalyst have been studied. In contrast to the recently established associative mechanism with a terminal alkene, where a neutral olefin adduct is formed, NBE reacts directly with the catalyst via [2 + 2] cycloaddition to form molybdacyclobutane with a reaction barrier about 30 kJ mol^–1^ lower in free energy than via the formation of a catalyst–monomer adduct. However, the direct cycloaddition of NBE was only found for one out of four stereoisomers. Our findings strongly suggest that this stereoselective approach is responsible for *E*-selectivity and point toward a substrate-specific reaction mechanism in olefin metathesis with neutral Mo imido alkylidene *N*-heterocyclic carbene bistriflate complexes.

## Introduction

**Scheme 1:**
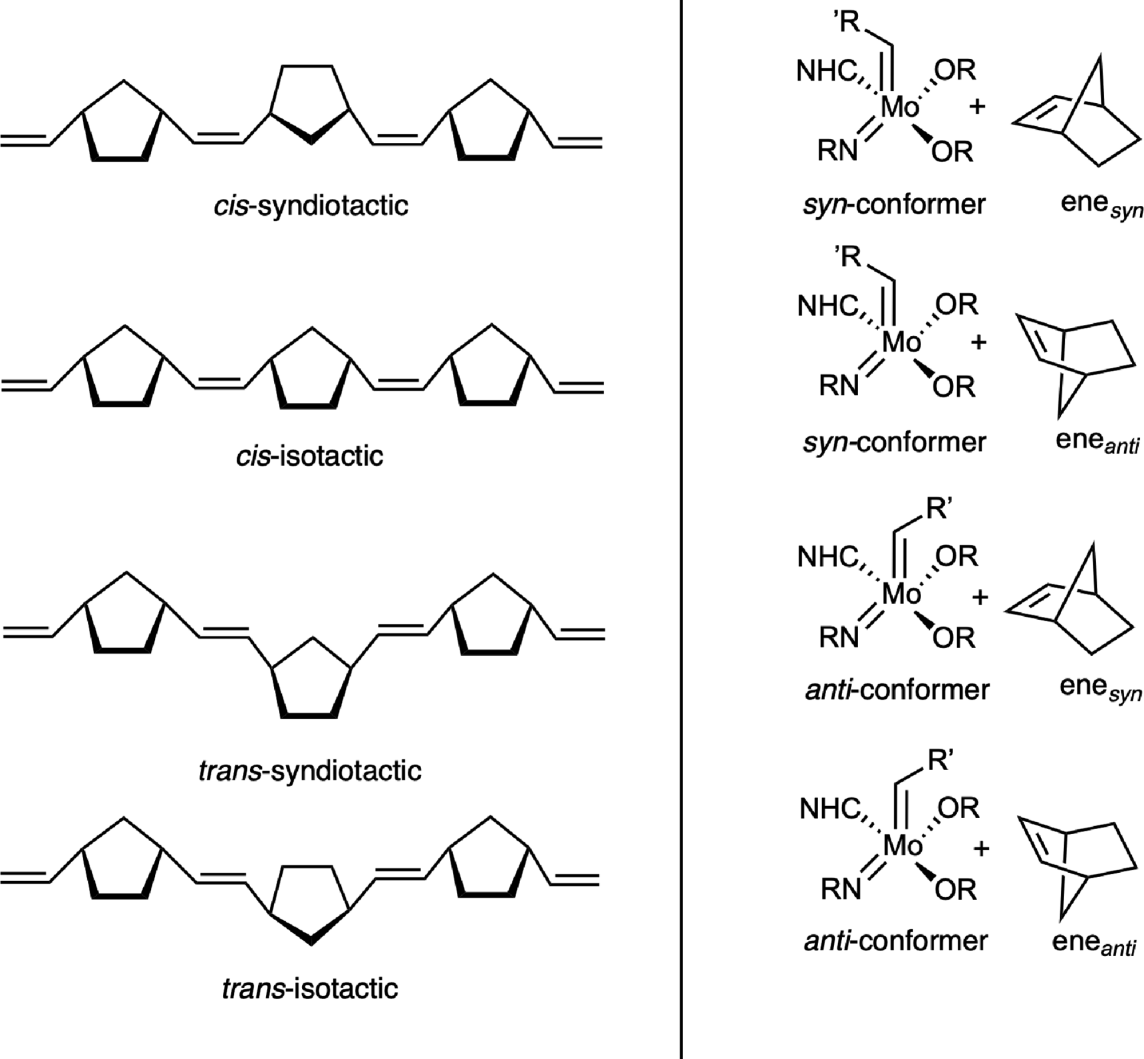
(left) Four Possible Regular Polymer Structures Formed from Norborn-2-ene via Ring-Opening Metathesis Polymerization. (right) Four Different Stereoisomeric Cycloadditions of NBE toward a Mo Imido Alkylidene NHC Catalyst

Olefin metathesis has become a key reaction in the formation of carbon–carbon bonds.^1−4^ The success can be attributed to the development of Mo- and W-based Schrock and Ru-based Grubbs initiators, which allow for such selective C–C coupling reactions under mild conditions and provide access to well-defined polymers with tunable properties.^3,5−8^ Although more reactive, the fast deactivation of Mo-based catalysts in comparison to Ru-based catalysts prevented so far their broader use.^9^ By contrast, neutral, pentacoordinated and cationic, tetracoordinated Mo imido alkylidene *N*-heterocyclic carbene (NHC) complexes show improved stability not only toward oxygen, water, and protic substrates but also toward high temperatures without sacrificing reactivity and productivity.^10−13^ Indeed, these catalysts are characterized by an impressive functional group tolerance and allow for olefin metathesis reactions with hydroxyl-, carboxyl-, aldehyde-, ether-, and amine-containing substrates.^14^ Apart from their high activity^15,16^ they also show remarkable stereoselectivity in ring-opening metathesis polymerization (ROMP), allowing the synthesis of highly tactic polymers.^17−20^ Generally, ROMP offers access to single-structure, functional oligomers or polymers.^8,18,19,21−25^ In that regards, controlling the stereoselectivity in ROMP is an ultimate driving force in catalyst design because the tacticity of a polymer is intricately linked to its properties.^23^ In the case of norborn-2-ene (NBE), four different regular polymers can be formed (see Scheme 1 (left)); these are *cis*-syndiotactic (*cis*-st), *cis*-isotactic (*cis*-it), *trans*-syndiotactic (*trans*-st), and *trans*-isotactic (*trans*-it). Typical Mo-based olefin metathesis catalysts can adapt a *syn-* or an *anti-*conformation, hence their interaction with NBE, that in turn can also be oriented in an ene_*syn*_ or ene_*anti*_ fashion toward the catalyst, resulting in four stereoisomeric reaction pathways as illustrated in Scheme 1 (right). Notably, although NBE is a rather reactive substrate, a recently reported Ru-based catalyst was found to be inert against ROMP of NBE in the presence of terminal olefins, once more illustrating the broad tunability of olefin metathesis catalysts.^26^

Formation of *cis*-st polymers can be rationalized by stereogenic metal control, where the metal stereocenter changes its conformation in each step;^22−24,27−29^*cis*-it polymers can be obtained by enantiomorphic site control.^22,23,28,29^

The synthesis of polymers that had a 92% *trans*-it base has been reported by Flook *et al*., though only for one monomer and proposedly proceeds via an ene_*anti*_ approach of the monomer to the *syn*-isomer of the initiator followed by turnstile rearrangement at the molybdacyclobutane.^27^ Very recently and with the aid of neutral and cationic Mo imido NHC alkylidene complexes, respectively, polymers with a ≥98% *trans*-it base have been prepared with a wide variety of monomers.^18^ Finally, formation of *trans*-st structures has been proposed to occur under chain-end control either via ene_*syn*_ addition of the monomer to the *anti*-conformer of the catalyst or ene_*anti*_ addition of the monomer to the *syn*-conformer of the catalyst.^5^

**Scheme 2:**
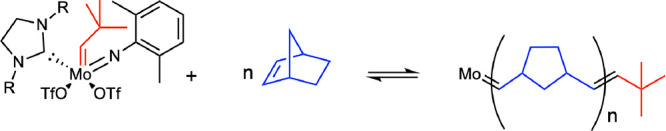
ROMP of NBE with Mo(*N*-2,6-Me_2_-C_6_H_3_)(CHCMe_3_)(MesH_2_)(OTf)_2_ (**1**, R = Mes = 2,4,6-(CH_3_)_3_C_6_H_2_, OTf = CF_3_SO_3_); Adapted from Ref (14)

Based on the experimental observation that in the ROMP of NBE and NBE-derivatives with Mo(*N*-2,6-Me_2_-C_6_H_3_)(CHCMe_3_)(IMesH_2_)(OTf)_2_ predominantly *trans*-configured polymers have been obtained (Scheme 2),^14^ we herein report quantum chemical investigations on both the different reaction pathways and the *E*-selectivity in the first reaction cycle. Quantum chemical methods, in particular, density functional theory, have successfully been used in the past to elucidate the reactivity and reaction mechanisms of various olefin metathesis catalysts.^30−39^

In a previously combined experimental and quantum chemical study, we already reported on the reaction mechanism of neutral and cationic Mo imido alkylidene NHC catalysts in the metathesis reaction with 2-methoxystyrene.^40^ Both kinetic measurements and our DFT studies strongly suggest that the reaction of 2-methoxystyrene with neutral Mo imido alkylidene NHC catalysts proceeds in an associative fashion, during which a neutral olefin adduct is formed. The catalytic cycle is then initiated by dissociation of one triflate and generation of the cationic catalyst, followed by cycloaddition of the substrate to form a molybdacyclobutane intermediate and, finally, cycloreversion. In line with experiments, the rate-determining step is the cycloaddition. Remarkably, a reassociative “S_N_2-type” pathway describing a substrate-induced triflate dissociation was found to be similar in energy to the associative one. In contrast, a fully dissociative pathway, in which one triflate dissociates prior to substrate coordination, contradicts experimental findings. However, in any case, olefin metathesis itself starts upon formation of the cationic species.

Here, we report on the detailed mechanistic investigation of the *E*-selective ROMP of NBE with Mo(*N*-2,6-Me_2_-C_6_H_3_)(CHCMe_3_)(IMesH_2_)(OTf)_2_. Our quantum chemical studies shed light on the origins of this *E*-selectivity in order to rationalize why predominantly *trans-*polymers are found with Mo(*N*-2,6-Me_2_-C_6_H_3_)(CHCMe_2_Ph)(IMesH_2_)(OTf)_2_. To obtain reliable computational results, it was not only essential to choose an adequate combination of density functional, basis set, and solvent model to calculate the electronic energy for a given structure but also, even more important, to identify the most stable conformer of a given species. This is particularly true for highly flexible transition metal complexes, where the generation of conformers still poses a challenge. For these purposes, the recently developed Conformer-Rotamer-Ensemble-Sampling Tool (CREST) was used to identify low-energy conformations, making use of improved semiempirical methods.^41,42^

## Computational Methodology

The single-crystal X-ray crystal structure of Mo(*N*-2,6-Me_2_-C_6_H_3_)(CHCMe_3_)(IMesH_2_)(OTf)_2_ reported in ref (14) served as an initial starting structure for our calculations. The IMesH_2_ moiety was modified to IMes (1,3-dimesitylimidazol-2-ylidene) to yield Mo(*N*-2,6-Me_2_-C_6_H_3_)(CHCMe_3_)(IMes)(OTf)_2_ denoted as **1**.

Density functional theory (DFT) has been the method of choice to investigate reaction mechanisms of organometallic catalysts due to its fast implementation and moderate computational costs.^43^ All structures were fully optimized using the BP86 density functional^44,45^ and the def2-SVP^46^ basis set on all atoms. In addition, effective core potentials of the SDD type were provided for Mo.^47^ Solvent effects were modelled implicitly with the conductor-like screening model (COSMO)^48,49^ as implemented in Turbomole with ε = 9.0 to account for 1,2-dichlorethane, which was included in the structure optimizations. Reported electronic energies were calculated as single points on the previously optimized structures using BP86-D3/def2-TZVP^46^/SDD in an implicit solvent, where empirical dispersion corrections of the D3 type with Becke–Johnson damping were invoked.^50,51^

Thermal and entropic corrections to the electronic energies were obtained at the BP86/def2-SVP/COSMO (ε = 9.0) level. Obtained frequencies were scaled with a factor of 1.0207,^52^ modes below 100 cm^–1^ were set to 100 cm^–1^ to minimize artifacts in the calculation of entropy,^53^ and the steady-state conversion to correct to the reference state of 1 mol L^–1^ was applied using the GoodVibes python script.^54^ Resulting values were added to the BP86-D3/def2-TZVP/SDD/COSMO (ε = 9) electronic energies and reported reaction free energies were calculated at 303 K.

Due to the high flexibility of the ligands in the products, a conformer search was performed with the Conformer-Rotamer-Ensemble-Sampling Tool (CREST) as developed by Grimme *et al*. to identify low-energy conformations.^41^ The idea of this approach is to use a cheap semiempirical electronic structure method (here, the DFT tight binding variant is GFN2-xTB^42^) in a metadynamics simulation,^55^ where the root-mean-square deviation (RMSD) to the reference structure serves as a collective variable to effectively sample the phase space. As the accuracy of describing transition metals is often compromised in semiempirical approaches,^56^ the most favorable xTB conformers may not necessarily coincide with the DFT ones (see also Figures S1–S4 in the Supporting Information). Hence, the CREST was used to generate large structural ensembles of xTB conformers with up to several hundred individual structures. Subsequently, the structures were aligned on the Mo center and the NHC ligand and a hierarchical clustering algorithm was applied with a cutoff of 2.5 Å to obtain between 15 and 25 representative cluster structures (see Table S1 of the Supporting Information for details). These were then optimized with BP86/def2-SVP/SDD/COSMO as outlined above. For all four stereoisomeric products, this procedure identified lower energy conformers (up to 30 kJ mol^–1^, Table S1) than the ones generated by chemical intuition. The CREST runs were started with the following settings: GFN2-xTB (−gfn2) was chosen as the semiempirical electronic structure method and solvent effects were modelled implicitly with a generalized Born model. Since no parameters for 1,2-dichloroethane were available, dichloromethane was chosen (−g CH_2_Cl_2_). The energy window was set to 30 kcal mol^–1^ (−ewin 30), that is, xTB conformers that lie within this energy range with respect to the most favorable conformer were accepted and discarded otherwise. The energy threshold between two conformers was set to 0.1 kcal mol^–1^ (−ethr 0.1) and the RMSD threshold to 0.1 Å (−rthr 0.1). If two conformers differed in energy by more than 0.1 kcal mol^–1^ and by more than to 0.1 Å in their RMSD, they were kept as separate conformers. This procedure has successfully been applied for the conformer generation of transition metal-containing complexes.^57,58^

Initial transition-state searches were performed by reaction path optimization as implemented in Turbomole^59^ by invoking the *woelfling* tool. Reactant and product structures are connected by the reaction path, which is discretized to yield *n* intermediate structures. Such methods are often referred to as “double-ended” searches. The only constraint here was that the structures are equally spaced; a quadratic potential prevented the intermediate structures from converging to the reactant or the product state and was applied in the optimization.^60^ In the Turbomole implementation, a modified linear synchronous transit algorithm was used.^61^ Energy maxima of these reaction paths were used as starting points for an eigenvector following to localize the transition state. Obtained converged structures were verified to be the desired transition states by analysis of the Hessian matrix, where in the case of a true transition state, only one imaginary frequency was found that coincides with the reaction coordinate. In some instances, no converged transition state could be located; hence, the energy maximum of the reaction path investigation was chosen as the approximate structure and indicated by an asterisk “*.” All calculations were performed using the quantum chemical program suite Turbomole.^59,62^ Structures were visualized using PyMol^63^ and reaction energy diagrams were obtained with Origin.^64^

Modifying IMesH_2_ to IMes, the Tolman electronic parameter decreases from 2051.0 to 2050.3 cm^–1^ and σ-donor strength increases, which in turn results in a decrease in the turnover frequency as recently reported for cationic Mo imido NHC alkoxide catalysts when undergoing ring-closing metathesis or cross metathesis.^12^ However, the conclusions drawn from this study are not affected by the modification of the NHC. Test calculations as listed in the Supporting Information in Tables S2 and S3 show minor differences and are in line with experimental findings.

## Results

**Figure 1:**
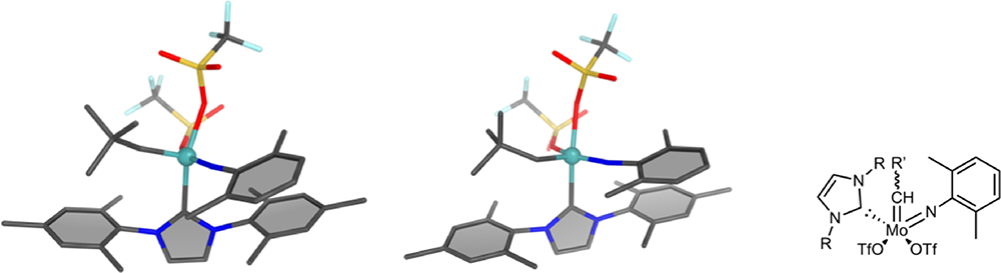
Molecular structure of catalyst **1** in its *anti*- (left) and *syn*-conformation (middle) and the Lewis formula (right).

**Scheme 3:**
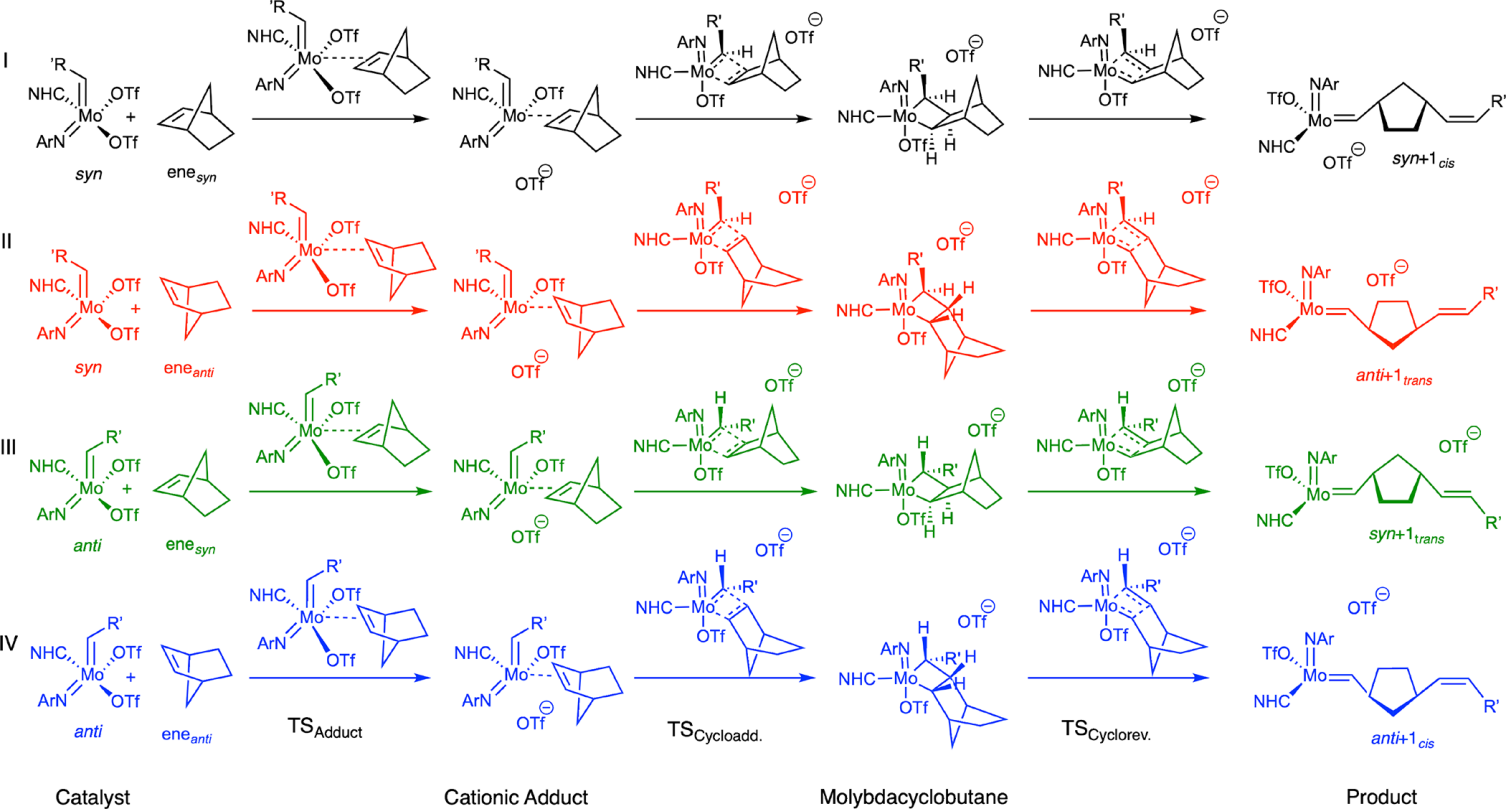
Schematic Reaction Mechanism of the *E*-Selective ROMP of NBE with **1** The catalytically active species is a cationic adduct structure, which undergoes cycloaddition to form the molybdacyclobutane ring, followed by cycloreversion to form the products; the four reaction pathways account for the two isomeric forms of **1** with the alkylidene group being either *syn* or *anti* and the two orientations of the substrate toward **1** being either ene_*syn*_ or ene_*anti*_.

We investigated the *E*-selective ROMP of NBE with **1** (see Figure 1) for the first reaction cycle. The four reaction routes **I**–**IV** are depicted in Scheme 3, in which the starting structures, relevant reaction intermediates, such as the cationic adduct and molybdacyclobutane, and the reaction products are shown. The different routes **I**–**IV** originated from the interaction of NBE either in an ene_*syn*_ or ene_*anti*_ fashion with the catalyst either in its *syn*- or *anti*-conformation (compare Scheme 1).

**Figure 2:**
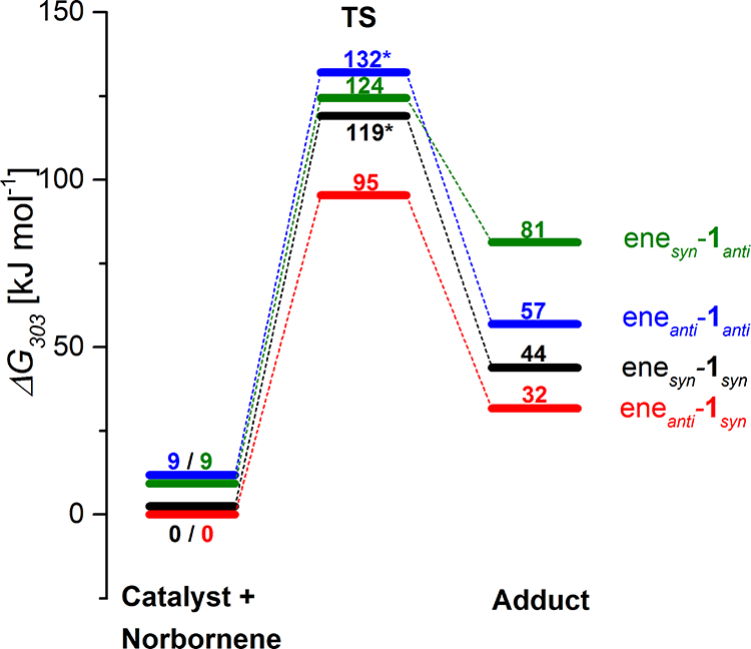
Relative reaction free energies (BP86-D3/def2-TZVP/SDD/COSMO (ε = 9.0)//BP86/def2-SVP3/SDD/COSMO (ε = 9.0)) in kJ mol^–**1**^ for the initial reaction step of NBE and the catalyst resulting in the cationic adduct, which forms an ion pair with the dissociated triflate. Catalyst **1** can either be in a *syn*- or in an *anti*-conformation with respect to the alkylidene and the NBE can interact with the complex in an ene_*syn*_ or an ene_*anti*_ orientation, resulting in four different adducts. The reaction barriers for ene_*syn*_-**1**_*syn*_ (black) and ene_*anti*_-**1**_*anti*_ (blue) are approximated by reaction path sampling as indicated by the asterisk, while the transition states for ene_*anti*_-**1**_*syn*_ (red) and the ene_*syn*_-**1**_*anti*_ (green) have been localized.

The relative reaction free energies for the initial reaction step are depicted in Figure 2. These Δ*G* values consist of BP86-D3/def2-TZVP/SDD/COSMO electronic energies, where the 1,2-dicholorethane solvent is modelled implicitly with the dielectric constant of ε = 9, plus thermodynamic and zero-point corrections calculated with BP86/def2-SVP/SDD/COSMO in implicit solvent (for details, see Computational Methodology).

Interestingly, the catalyst is more stable in its *syn*- than in its *anti*-conformation by Δ*G*_303_ = 9 kJ mol^–1^, but the energy difference is small enough that moderate amounts of *anti*-conformation are present in the reaction mixture. Remarkably, despite extensive searches, no neutral adducts of the catalyst and the NBE substrate were found for any of the four stereoisomers, which is in contrast to the mechanistic study reported for 2-methoxystyrene and the same catalyst.^40^ Rather, the substrate interacts with the catalyst in an “S_N_2-type” step, where coordination to the catalyst is accompanied by dissociation of the triflate *trans* to the alkylidene group. This distinct difference in reactivity between NBE and 2-methoxystyrene can be attributed to the bulkier nature of NBE and its high reactivity.

**Figure 3:**
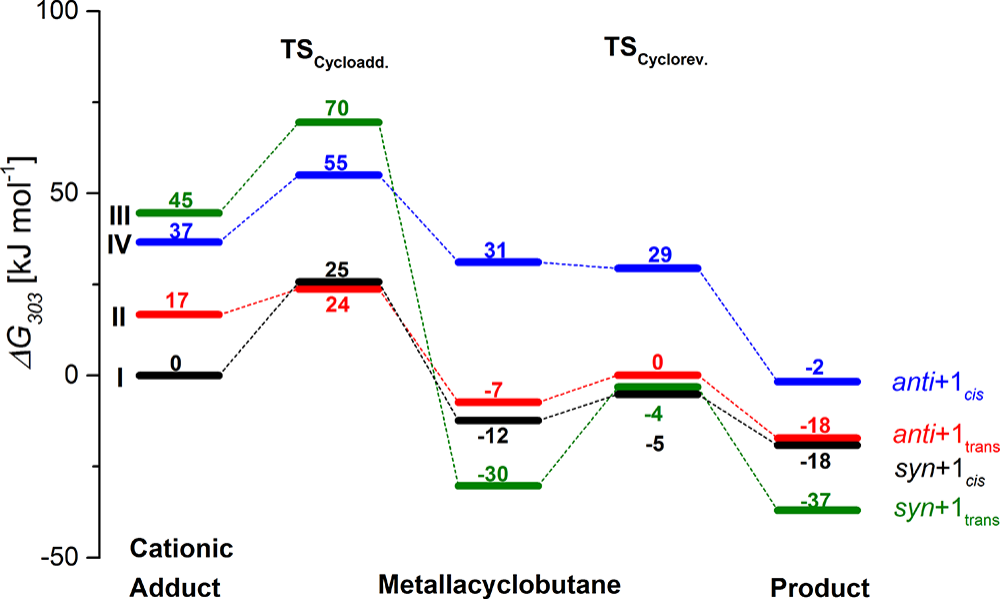
*E*-selective product formation starting from the catalytically active cationic adduct, which undergoes cycloaddition to the molybdacyclobutane ring, followed by cycloreversion yielding the products. All energies are reaction free energies Δ*G* given in kJ mol^–**1**^ calculated with BP86-D3/def2-TZVP/SDD/COSMO//BP86/def2-SVP/SDD/COSMO at 303 K.

The transition states for this step could only be fully characterized for the ene_*syn*_-**1**_*anti*_ (green) species and the ene_*anti*_-**1**_*syn*_ isomer (red), whereas for the two other species, the presented numbers are estimates derived from a reaction path optimization as implemented in Turbomole (see Computational Methodology for details).^59^ These estimated numbers are typically upper bounds for the true transition states. It can be seen that the energetically most favorable transition state is found for the ene_*anti*_-**1**_*syn*_ isomer (red) with a reaction barrier of Δ*G*^‡^_303_ = 95 kJ mol^–1^, followed by the ene_*syn*_-**1**_*syn*_ (black) one with a barrier height of Δ*G*^‡^_303_ = 119 kJ mol^–1^, and the ene_*syn*_-**1**_*anti*_ (green) one with a transition-state free energy of Δ*G*^‡^_303_ = 124 kJ mol^–1^ and a reaction barrier of Δ*G*^‡^_303_ = 115 kJ mol^–1^. The least favorable in both, the transition-state free energy and the barrier height, is the ene_*anti*_-**1**_*anti*_ (blue) isomeric route with an approximated reaction free energy of the transition state of 132 kJ mol^–1^ and a reaction barrier of 123 kJ mol^–1^. Considering the adduct structures, the two isomers, where the catalyst is in its *syn*-conformation, are more stable than those that is in its *anti*-conformation with *ene_syn_*-**1**_*anti*_ (green) being the least stable species. It is noteworthy here that each cationic adduct forms an ion pair with the dissociated triflate, which stabilizes the adduct as previously reported.^40^ As an adequate sampling of the triflate positions is challenging, the stabilization of the cationic species by formation of an ion pair with the dissociated triflate can, in a first approximation, be considered as a constant shift in the total energy. This finding is evident from the relative reaction free energies of the reaction intermediates in the presence of triflate as depicted in Figure S5, which by and large agree with those in Figure 3, and it is in agreement with previous investigations.^40^ Hence, to reduce complexity, we continued our investigations on the *E*-selectivity in ROMP with the cationic adduct species, which is known to be the catalytically active species in the absence of dissociated triflate.^40^

Relative Gibbs free energies at 303 K for the ring-opening pathway starting from the cationic NBE adducts to yield the four stereoisomers are depicted in Figure 3 (see also Scheme 3): the reaction path to the *anti* + 1*_cis_* product conformer is shown in blue (compare **IV** in Scheme 3), the one to the *anti* + 1_*trans*_ conformer in red (compare **II** in Scheme 3), the one to the *syn* + 1*_cis_* conformer in black (compare **I** in Scheme 3), and the one to the energetically most stable *syn* + 1_*trans*_ conformer in green (compare **III** in Scheme 3).

Starting from the relative stabilities of the cationic adducts, it can be seen that two adducts **I** and **II** with the catalyst in its *syn*-conformation and the NBE in its ene_*syn*_ and ene_*anti*_ orientations, depicted in black and red in Figure 3 (compare **I** and **II** in Scheme 3), are more stable than the two adducts with the catalyst in its *anti*-conformation. The ene_*syn*_ orientation of the substrate and the catalyst in the *anti*-conformation forms the least stable adduct **III** (green) here, with a relative free energy of Δ*G*_303_ = 45 kJ mol^–1^ compared to the most stable adduct **I** (black), whose energy has been arbitrarily set to zero. Analyzing the cationic adduct structures (Figures S20–S23, Supporting Information.), the ones for **I** (black), **II** (red), and **IV** (blue) are largely similar: the olefin unit of the NBE and the Mo-alkylidene bond form a small dihedral angle, thus are almost perfectly positioned for the cycloaddition, and show a Mo-C_NBE1_ η^1^-coordination (Figures S20, S21, and S23). In adduct **III** (green) as depicted in Figure S22, however, the olefin unit of NBE is almost parallel to the Mo-imido bond. Consequently, the Mo-C_NBE1_ and Mo-C_NBE2_ bond lengths differ by only 0.15 Å. Yet, adduct **III** still shows a tendency towards an η^1^-coordination.

The cationic NBE adducts can undergo [2 + 2] cycloaddition to form the molybdacyclobutane ring. The energy barriers associated with this reaction step are between 19 < Δ*G*^‡^_303_ < 25 kJ mol^–1^ with the notable exception of the cycloaddition barrier of the ene_*anti*_ orientation and the catalyst in *syn*-conformation **II** (red, compare Scheme 3); there, the barrier is ca. 7 kJ mol^–1^ and thus significantly lower.

Molybdacyclobutane shows a high stability for all four conformers and the reaction is exergonic for all four species. Remarkably, the molybdacyclobutane **III** (green) is the most stable conformer at Δ*G*_303_ = −30 kJ mol^–1^, despite originating from a catalyst in an *anti-*conformation, followed by molybdacyclobutane **I** and **II** (black and red) with the catalyst in the *syn*-conformation. Although a conformer search was performed for molybdacyclobutane **IV**, the identified most stable conformer is still significantly less stable than the other metallacylobutanes. To shed light on the extraordinary high stability of molybdacyclobutane **III**, we investigated the intramolecular noncovalent interactions in all molybdacyclobutanes (Figures S8–S11) by projecting the second eigenvalue of the electron-density Hessian matrix sign(λ_2_)ρ onto an isosurface of the reduced gradients with the NCIPLOT tool (see the Supporting Information for details).^65,66^ While all species showed attractive intramolecular interactions between the NHC and the NBE moieties, respectively, and the rest of the catalyst, for molybdacyclobutane **III**, we found methyl hydrogens and the NHC’s phenyl rings at distances of 2.65 and 2.76 Å (compare Figure S10) allowing for additional stabilizing, noncovalent CH−π interactions.^67^ These interactions are absent in all other molybdacyclobutane isomers.

The reaction barrier for cycloreversion is comparable or lower than that for the cycloaddition, depending on the conformer. A barrier of Δ*G*^‡^_303_ = 26 kJ mol^–1^ was found for the formation of the *syn* + 1_*trans*_ species (green), whereas barriers of Δ*G*^‡^_303_ ≈ 7 kJ mol^–1^ were determined for the formation of the *anti* + 1_*trans*_ and the *syn* + 1*_cis_* products (red and black). For the formation of the *anti* + 1*_cis_* product (blue), the barrier is only 4 kJ mol^–1^ in electronic energy. Because of the approximate calculation of thermodynamic corrections the free difference in free energy is even slightly negative, which is of course an artifact due to the approximate calculation of thermodynamic and zero-point energy corrections. Looking at the product stability, the *syn* + 1_*trans*_ species (green) is by far the most stable product with Δ*G*^‡^_303_ = −37 kJ mol^–1^, again followed by the *anti* + 1_*trans*_, and the *syn* + 1*_cis_* products with relative stabilities of Δ*G*^‡^_303_ = −18 kJ mol^–1^, whereas the *anti* + 1*_cis_* one is the least stable of all. Due to their inherent flexibility, the most stable product structures were only identified after extensive exploration of the phase space with the CREST conformer generator provided by Grimme *et al.*(41) and subsequent clustering and reoptimization of the structures (see Computational Methodology for details). Only with these low-energy conformers, the cycloreversion was found to be exergonic.

**Scheme 4:**
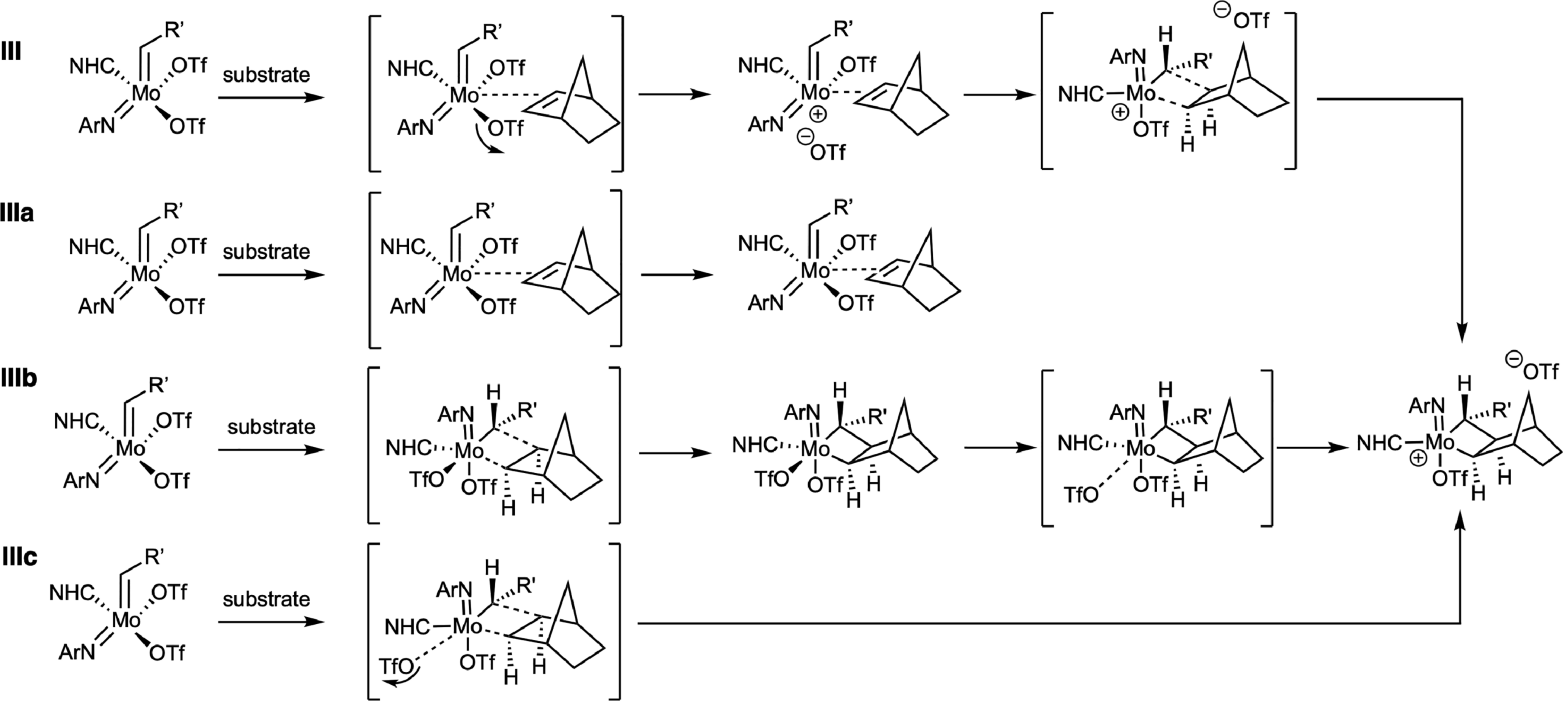
Alternative Reaction Pathways for the Ene_*syn*_ Interaction of NBE with the *anti*-Conformation of the Catalyst to Yield the Molybdacyclobutane Intermediate **III**: formation of the cationic adduct in an “S_N_2-type” pathway, followed by cycloaddition; **IIIa**: formation of a neutral olefin adduct in an associative pathway (not observed here); **IIIb**: direct associative formation of the neutral molybdacylcobutane ring followed by dissociation of triflate to yield the catalytically active cationic species; **IIIc**: direct “S_N_2-type” formation of the cationic molybdacyclobutane ring under simultaneous dissociation of triflate.

Interestingly, in agreement with the experiment,^14^ the *syn* + 1_trans_ product was found the be the most stable of all stereoisomers. However, the preceding adduct is the least stable of all and only at the molybdacyclobutane stage that this stereoisomer becomes energetically favored. This finding prompted us to look into the reaction pathway **III** in more detail and to investigate alternative mechanisms for the initiation of the reaction and for the formation of the molybdacyclobutane ring as depicted in Scheme 4.

**Figure 4:**
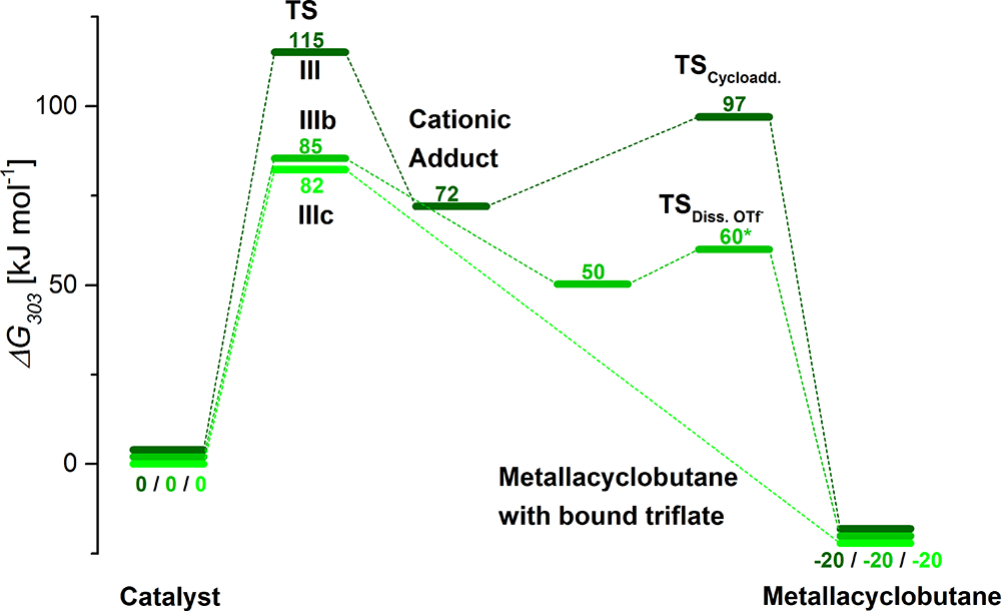
Catalytic pathways for NBE in the ene_*syn*_ orientation and catalyst **1** in its *anti*-conformation. Rather than the formation of a cationic adduct depicted in dark green (**III**), the reaction may also proceed via direct formation of the (neutral) molybdacyclobutane with bound triflate (green, **IIIb**) and subsequent dissociation of triflate or via direct formation of a (cationic) molybdacylcobutane and simultaneous ejection of triflate (light green, **IIIc**). All energies are reaction free energies Δ*G* given in kJ mol^–**1**^ calculated with BP86-D3/def2-TZVP/SDD/COSMO//BP86/def2-SVP/SDD/COSMO at 303 K. The asterisk indicates that the dissociation energy is approximated from the reaction path.

For the already investigated “S_N_2-type” initiation reaction (**III** in Scheme 4), in course of which the cationic adduct is formed directly under ejection of triflate, we found a significant barrier of Δ*G*^‡^_303_ = 115 kJ mol^–1^ associated with the transition state TS_Adduct_. Its reaction free energy is depicted in Figure 2 (green) and Figure 4 (dark green), respectively. Despite all efforts and in contrast to previous findings for the 2-methoxystyrene substrate, a reaction pathway yielding a neutral NBE adduct (pathway **IIIa** in Scheme 4) could not be found. All attempts to converge such a neutral adduct species failed for the bulky NBE. However, a direct associative cycloaddition transition state (TS_Cycloadd.-Associative_) was localized, where a neutral molybdacyclobutane with both triflates still attached to the metal is formed (**IIIb**, Scheme 4 and Figure 4). This reaction step is associated with a barrier of Δ*G*^‡^_303_ = 85 kJ mol^–1^, about 30 kJ mol^–1^ lower than for the formation of the cationic adduct. For the subsequent dissociation of triflate to form the cationic molybdacylcobutane ring, a low reaction barrier of Δ*G*^‡^_303_ ≈ 10 kJ mol^–1^ was estimated. The reaction was found to be exergonic with a free energy, for this step, of Δ*G*_303_ = −70 kJ mol^–1^, indicating that a neutral molybdacyclobutane is significantly less stable than the cationic species. Lastly, a third reaction route **IIIc** was identified, where the catalyst and NBE directly react under cycloaddition and dissociation of one triflate to form the cationic molybdacyclobutane. The barrier for this “S_N_2-type” route (TS_Cycloadd.-SN2_) was found to be very similar to the previous one at Δ*G*^‡^_303_ = 82 kJ mol^–1^.

**Figure 5:**
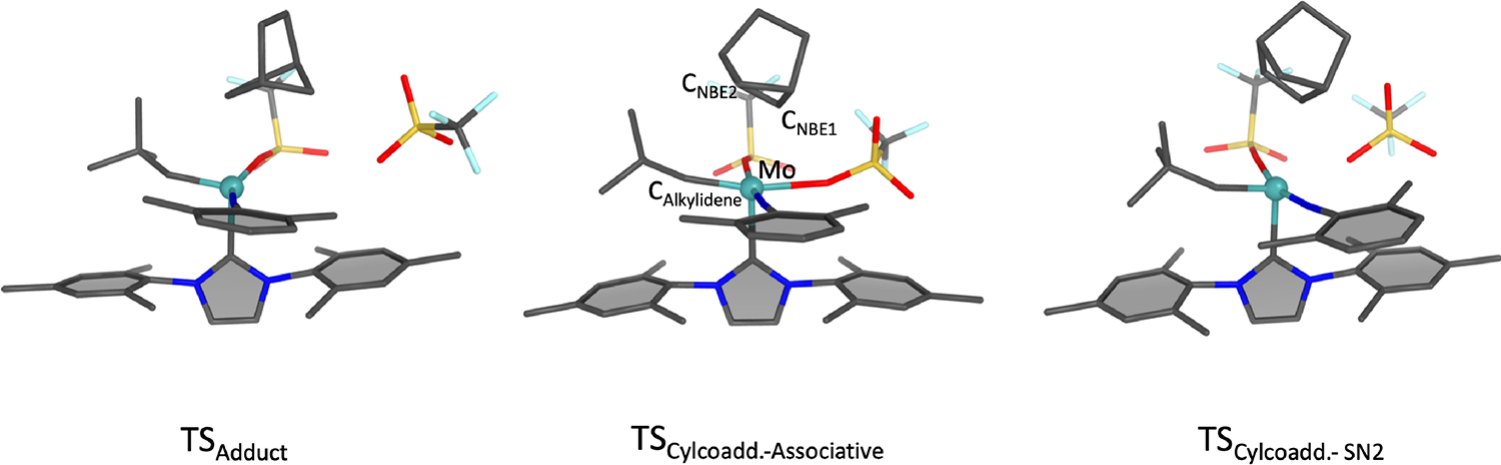
Comparison of the various transition states for an ene_*syn*_ approach of NBE to the catalyst in its *anti*-configuration. Left: transition state TS_Adduct_ for the formation of the cationic adduct. Middle: transition state TS_Cycloadd.-Associative_ for the associative cycloaddition to form the neutral molybdacyclobutane. Right: transition state TS_Cycloadd.-SN2_ for the “S_N_2-type” cycloaddition to form the cationic molybdacyclobutane under simultaneous dissociation of triflate.

Comparing the structures of these three transition states (see Figure 5), one can see that in TS_Adduct_ (pathway **III** in Scheme 4 and Figure 4), the olefin unit of NBE is almost parallel to the Mo-imido bond with Mo-C_NBE1_ and Mo-C_NBE2_ distances of 3.12 and 3.13 Å, respectively, while the triflate is already at a Mo-O_triflate_ distance of 4.43 Å. The two other transition states, TS_Cycloadd.-Associative_ (pathway **IIIb** in Scheme 4 and Figure 4) and TS_Cycloadd.-SN2_ (pathway **IIIc**, Scheme 4 and Figure 4), resemble each other: the Mo-C_NBE_ bond length is 2.63 Å and the C_alkylidene_-C_NBE2_ is 3.17 Å in TS_Cycloadd.-Associative_, whereas they are somewhat longer in TS_Cycloadd.-SN2_ with *d*_Mo-C(NBE1)_ = 2.80 Å and *d*_C-alkylidene-C(NBE2)_*=* 3.61 Å. The Mo-O_triflate_ distance is 2.86 Å in TS_Cycloadd.-SN2_, but also in TS_Cycloadd.-Associative_, it is elongated by about 0.2 Å compared to **1** in which *d*_Mo-O(triflate)_ = 2.41 Å. This finding indicates a bond activation, which is also supported by the low-lying transition state for triflate dissociation (compare Figure 4).

## Discussion

### Computational Protocol

To calculate reliable reaction energies, it was essential to perform conformer searches on the highly flexible product structures to identify the most stable species with computationally affordable semiempirical methods (here: GFN2-xTB). By standard modelling based on chemical intuition, we were not able to identify the most stable conformer. We would have incorrectly predicted the molybdacyclobutane to be thermodynamically more favorable than the product. Despite major progress,^42^ the accuracy of semiempirical methods is compromised due to the invoked approximations and the use of a minimal basis set.^56,68^ While we found a good agreement for the geometries when comparing the GFN2-xTB-optimized conformers to the BP86 ones, their energy ranking differed significantly (compare Figures S1–S4 of the Supporting Information). This finding led us to pool all obtained conformers, cluster them, and use cluster representatives for further quantum chemical investigations. Such an approach has been successfully applied for Zn-containing complexes.^57^ In addition, this protocol was recently benchmarked for a number of transition metal clusters including Mo complexes and was shown to reliably identify low-energy conformers.^58^ Another aspect of the computational protocol concerns the treatment of the dissociated triflate that forms an ion pair with the cationic catalyst species. A previous study showed that on the one hand, an explicit description of the ion pair in a supermolecular approach was necessary to obtain consistent reaction free energies for the entire reaction pathway, a finding supported by experimental data, where in NMR studies the dissociated triflate was found to be “nearby”.^40^ On the other hand, this study also indicated that the stabilization of the cationic species by complexation with the dissociated triflate could be roughly considered as a constant shift in energy in total energy.^40^ Hence, the explicit treatment of triflate in a supramolecular approach can be omitted if one is only interested in comparing the reaction pathway(s) once the catalytically active reaction species is formed. Compared to the experimental reaction conditions, these two scenarios are the extreme cases. It is likely that in the liquid phase, the dissociated triflate is to some degree shielded by the solvent weakening the impact on the catalytic species. In the presented results here for the ROMP of NBE, we see by comparing the reaction free energies in Figures 2 and 3 that the stability of the ene_s*yn*_-**1**_*syn*_ adduct vs the ene_*anti*_-**1**_*syn*_ depends on whether the dissociated triflate is considered (Figure 2) or omitted (Figure 3). Comparing the relative reaction free energies of the reactants, intermediates, and products in the absence of (explicitly treated) triflate (Figure 3) and when forming an ion complex (Figure S5), we see, however, that the results are similar. Whether or not the dissociated triflate is incorporated in the calculation does not affect the conclusions that can be drawn from these investigations. Of course, the extent to which an intermediate is stabilized by the formation of an ion pair may be inherent to the individual species. However, the most remarkable differences are found for the products, where recoordination of the dissociated triflate stabilizes the *syn* + 1_trans_ species relative to all other stereoisomers, further driving selectivity.

### Reaction Mechanism

Analysis of the reaction mechanism disclosed that ROMP of NBE with a Mo imido NHC alkylidene catalyst proceeds via the known cycloaddition, metallacyclobutane formation, cycloreversion scheme proposed by Hrisson and Chauvin.^69^ While a recent study revealed the formation of an unprecedented neutral olefin adduct as an initial reaction step for this class of catalyst, no neutral catalyst–monomer adduct was found for NBE. Its bulkiness and high reactivity in comparison to 2-methoxystyrene^40^ inhibit a fully associative pathway here. In fact, our previous reported calculations with the less bulky *t*-butylethylene substrate already showed that a formation of the neutral olefin–catalyst adduct is no longer possible.^40^ Instead, the NBE-induced triflate dissociation in an “S_N_2-type” fashion as determined in this study becomes the main pathway. Interestingly, this mechanism was found to be a secondary pathway for 2-methoxystyrene with a slightly higher reaction barrier.^40^ Consequently, analysis of the reaction mechanisms of NBE and 2-methoxystyrene strongly points toward a substrate-specific reaction path, while the cycloaddition is in any case the rate-determining step.

### *E*-Selectivity

In line with experimental findings, which revealed 85–90% *E*-selectivity in the ROMP of simple NBE-derivatives by the action of Mo(*N*-2,6-Me_2_-C_6_H_3_)(CHCMe_3_)(IMesH_2_)(OTf)_2_,^14^ the thermodynamically most stable product structure (**III**), originating from an ene_*syn*_-**1**_*anti*_ approach, has a *trans*-double bond. Product **II**, originating from an ene_*anti*_-**1**_*syn*_ approach, also with a *trans-*double bond, and product **I**, originating from an ene_*syn*_-**1**_*syn*_ approach, with a *cis-*double bond, are both ca. 19 kJ mol^–1^ less stable than the former. However, no significant amounts of *cis*-product are expected to be formed because the formation of adduct **I** has a barrier of 119 kJ mol^–1^ (Figure 2) and is, thus, kinetically hindered. The stability of the products with *trans*-double bonds is even more pronounced when the dissociated triflate recoordinates (Figure S5). While the analysis of the product stability is promising to explain the *E*-selectivity, a closer look at the reaction mechanism reveals a different picture: First, based on the free-energy difference of 9 kJ mol^–1^ between **1**_*syn*_ and **1**_*anti*_, we would expect a concentration of the *anti*-conformer of 3 to 13%, assuming chemical accuracy of the quantum chemical results (<4 kJ mol^–1^). This is enough to react with NBE yet too little to explain the selectivity. However, Mo-based catalysts are known to show fast *syn*–*anti* interconversion.^70^ In fact, for a series of Mo–alkoxide catalysts, Oskam and Schrock reported a fast interconversion of the stereoisomers as determined by NMR studies.^70^ If interconversion is faster than metathesis, the initially low *anti* content does not matter because the s*yn*- and the *anti*-conformers rapidly equilibrate. The stereoselectivity then only depends on the differences in reaction barriers of the rate-determining step (ΔΔ*G*) for the various stereoisomeric routes according to the Curtin–Hammett principle. Oskam and Schrock also found that the less electron-withdrawing the alkoxide ligand is, the faster the rate of interconversion. As the NHC ligand in **1** is a sigma-donor that effectively stabilizes the positive charge of Mo by donating an electron,^12^ it can be assumed that the rate of *syn*–*anti* interconversion is of similar rate or faster than metathesis.

Second, not only the formation of the cationic adduct **III** has a higher reaction barrier than the cationic adduct **II** (see Figure 2) but also adduct **III** is the least stable of all four species (see Figure 3). Hence, the established olefin metathesis mechanism does not seem to provide a conclusive answer to *E*-selectivity. Instead, by investigation of alternative reaction routes, we found that the ene_*syn*_-**1**_*anti*_ isomer (**III**) can undergo direct cycloaddition of NBE to the neutral catalyst. This alternative reaction pathway has a reaction free-energy barrier about 30 kJ mol^–1^ lower in energy than the one found for the formation of the cationic adduct (Figure 4). The lower energy is a direct consequence of the fact that no energetically “unfavorable” cationic adduct needs to be formed. The cycloaddition may either take place via addition of NBE to **1** to form the neutral molybdacyclobutane followed by triflate dissociation (route **IIIb**, Scheme 4 and Figure 4) or via an “S_N_2-type” reaction to form the cationic molybdacyclobutane directly (route **IIIc**, Scheme 4 and Figure 4). By comparing the structures of the transition states TS_Cyloadd.-Associative_ and TS_Cyloadd.-SN2_, one can see that the (partial) dissociation of the triflate in TS_Cyloadd.-SN2_ resulted in an earlier, that is, more “reactant-like”, transition state indicating that the Mo center becomes more reactive when triflate is about to dissociate. However, based on the very similar energies for these reaction routes, we cannot exactly determine which one is predominant, in particular, because the Mo-O_triflate_ bond is already activated in the neutral molybdacyclobutane and the barrier to dissociation is very low Δ*G***^‡^**_303_ ≈ 10 kJ mol^–1^. In view of these subtle differences, the true mechanism might well be in between associative and “S_N_2-type” but definitely not dissociative.

Our calculations clearly show that the reaction mechanism to form the first insertion product is stereospecific: a direct molybdacyclobutane formation is found for the ene_syn_-**1**_*anti*_ approach (green) and an adduct formation in all other instances. However, the difference in the reaction barrier of the rate-determining step for the two most favorable stereoisomeric routes to yield the first insertion products *syn* + 1_*trans*_ (green) and *anti* + 1_t*rans*_ (red) is rather small. Although formation of *syn* + 1_*trans*_ (green) is kinetically favored, the differences might be within the error margin of DFT, consequently, some *anti* + 1_t*rans*_ (red) could be formed. Thermodynamics on the other hand, clearly favor the formation of *syn* + 1_*trans*_ (green) (Figure 3). Therefore, *syn*–*anti* conversion may also occur at the first insertion product stage, despite the reaction mechanism being unknown, which may be another factor driving the selectivity.

Direct cycloaddition has been tested for all other stereoisomers, but the corresponding transition states could, to the best of our efforts, not be localized. It seems that this direct molybdacyclobutane formation is only possible for an ene_*syn*_ approach of the monomer to the catalyst in an *anti*-conformation (route **III**). Manual exploration of the molybdacyclobutane potential energy surfaces showed that small perturbations of the ring structures, e.g., elongation of the Mo-C_NBE1_ and C_alkylidene_-C_NBE2_ distances by 0.3 Å, resulted in a formation of cationic adducts (**I**, **II**, and **IV**). For the route **III**, analysis of the transition state TS_Cycloadd.-SN2_ revealed Mo-C_NBE1_ and C_Alkylidene_-C_NBE2_ distances of 2.80 and 3.61 Å. This means that already at a significant distance of NBE to the catalyst, the formation of the molybdacyclobutane is energetically downhill, which further supports the high reactivity of NBE in the ene_*syn*_ orientation with **1**_*anti*_. Once more, these findings point toward significant differences in the mechanism for the different reaction routes.

The extraordinary high stability of molybdacyclobutane **III** can be attributed to favorable noncovalent intramolecular interactions: In particular, the arrangement of the alkylidene, the NHC, and the imido with distances of the methyl hydrogen to the aromatic rings at around 2.6 Å allow for stabilization of CH−π interactions.^67,71^ These noncovalent interactions, that are typically in the order from 6 to 10 kJ mol^–1^,^67^ are absent in all other ring conformers (compare Figures S8–S11) but are well known in asymmetric organic catalysis as driving forces for stereoselectivity.^72,73^

To further elucidate the *E*-selectivity as a prerequisite for the high *trans-*content of the polymer of **1**, we compared the results to a second catalyst, Mo(*N*-2,6-Me_2_-C_6_H_3_)(CHCMe_3_)(I-*t*Bu)(OTf)_2_ (**2**, I-*t*Bu = 1,3-di-*t*-butyl-1,3-dihydro-2H-imidazol-2-ylidene, (compare Figure S6)). Compound **2** differs from **1** only in the NHC but shows a significantly lower *trans*-content in NBE-based polymers.^14^

The first striking discrepancy between the two catalysts is that the relative energy difference between the *syn*- and *anti*-conformations of **2** is 31.7 kJ mol^–1^, more than three times larger than that of **1**, decreasing the presence of the *anti-*conformer from 2.5% (**1**) to 3.3 × 10^–4^% (**2**). While the relative stabilities of the cationic adducts and the products of **2** are largely similar to those of **1** (compare Figure S7), significant differences are found for the molybdacyclobutanes. For **2**, they are more stable than the products (despite extensive conformer searches), with molybdacyclobutane **II** originating from the *ene_anti_*-**2**_*syn*_ approach being the energetically most favorable species overall. Again, the high stability of this metallacyclobutane can be attributed to additional aromatic CH−π interactions (Figure S12) that are absent in the other molybdacyclobutane isomers. Given the distinct differences between the two catalysts **1** and **2**, it is difficult to pinpoint the origin of the lower *trans*-content of the resulting polymer of **2**, but it may result as a consequence of all of these factors.

In the present study, we investigated the first reaction cycle because the activation process is an important step towards explaining the *E-*selectivity. Of course, for a complete explanation of the *E*-selectivity, the reaction between norbornene and the first insertion product(s) should be considered too. As the alkylidene of the insertion product differs from neopentylidene, some differences in the reaction energetics can be expected. However, due to the high flexibility of the alkylidene moiety in the insertion product, reliable modelling is extremely challenging.

In summary, we propose that the high *trans-*content of NBE-based polymers prepared by the action of **1** is a result of the energetically favorable direct stereoselective cycloaddition and favorable intramolecular noncovalent interactions that are only present in molybdacyclobutane **III**. Fast *syn*–*anti* interconversion at the catalyst or the product stage may further drive the selectivity.

## Conclusions

Our quantum chemical studies revealed that *E*-selectivity in the ROMP of NBE with neutral Mo imido alkylidene NHC complexes most likely originates from a direct cycloaddition of NBE to form molybdacyclobutane, while fast *syn*–*anti* interconversion may be an additional driving factor. Still, the favorable direct [2 + 2] cycloaddition was only found for one out of four stereoisomeric routes and is in contrast to findings for 2-methoxystyrene, indicating a substrate dependence of the reaction mechanism. This substrate dependence illustrates the difficulty in quantum chemical modelling to validate the proposed reaction mechanism by checking various substrates. Comparison with a second less *trans-*selective catalyst suggests that the stereoselectivity for ROMP of NBE with **1** may arise from multiple factors and an intricate interplay of catalyst and substrate. However, full characterization of the *E*-selectivity would require investigation of the second reaction cycles and is beyond the scope of this work.

Conformer generation of the product structures was necessary to identify the most stable conformers and to correctly predict the reaction energies. This finding emphasizes the need to include such routines in state-of-the-art modelling in quantum chemistry to increase accuracy. Only such high accuracy will allow us to understand and predict *E*/*Z*-selectivity in ROMP in the future.

## Supporting Information Available

Computational methodology to generate conformers with CREST and to calculate noncovalent interactions; additional reaction energy profiles, visualization of noncovalent interactions, structures of all investigated species (PDF)
Cartesian coordinates of all calculated structures are listed in “om1c00229_si_002.xyz” (XYZ)

## Supplementary Material

om1c00229_si_001.pdf

om1c00229_si_002.xyz
