# Supplementary material for: On the Origin of E-Selectivity in the Ring-Opening Metathesis Polymerization with Molybdenum Imido Alkylidene N-Heterocyclic Carbene Complexes
Source: Organometallics. 2021 Jul 9;40(15):2478–88. doi: 10.1021/acs.organomet.1c00229 (PMC8356225; doi:10.1021/acs.organomet.1c00229)
Supplement: Supplementary file 1 — om1c00229_si_001.pdf [file om1c00229_si_001.pdf]

Supporting Information

On the Origin of *E*-Selectivity in the Ring-Opening Metathesis Polymerization with Molybdenum Imido Alkylidene *N*-Heterocyclic Carbene Complexes

Maren Podewitz,[1] Suman Sen[2] Michael R. Buchmeiser*[2]*

[1]Institute of General, Inorganic and Theoretical Chemistry, and Centre of Molecular Biosciences, University of Innsbruck, Innrain 80/82, 6020 Innsbruck, Austria.

[2]Institute Institute of Polymer Chemistry and Institute of Organic Chemistry, University of Stuttgart, Pfaffenwaldring 55, D-70569 Stuttgart, Germany.

maren.podewitz@uibk.ac.at, michael.buchmeiser@ipoc.uni-stuttgart.de

Table of Contents

Computational Methodology 2
Reaction Energy Profiles 10
Visualization of Non-Covalent Interactions in Molybdacyclobutane Intermediates 13
Structures of the Investigated Species 19
References 38

Computational Methodology

Generation of Conformers

Due to the flexibility of the alkylidene with the opened norbornene in **products I-IV**, we performed extensive conformer search with the Conformer-Rotamer Ensemble Tool with subsequent hierarchical clustering and re-optimization with full DFT as described in the Computational Methodology.

The number of individual conformers as well as the number representative clusters obtained for each of the four product stereoisomers is listed in Table S1.

Table S1. Number of individual conformers as obtained from CREST runs, number of representative clusters, RMSD cutoff for the hierarchical clustering, and the gain in free energy compared to the initial conformer obtained by chemical intuition (in kJ mol^{-1}).

	# Crest Conformers	# Representative Cluster	Cutoff [Å]	Free Energy Gain[a]
Product I	396	23	2.5	-27.7
Product II	98	21	2.5	-14.0
Product III	211	25	2.5	-27.2
Product IV	113	15	2.5	-25.9

[a] In comparison to the conformer obtained by chemical intuition.

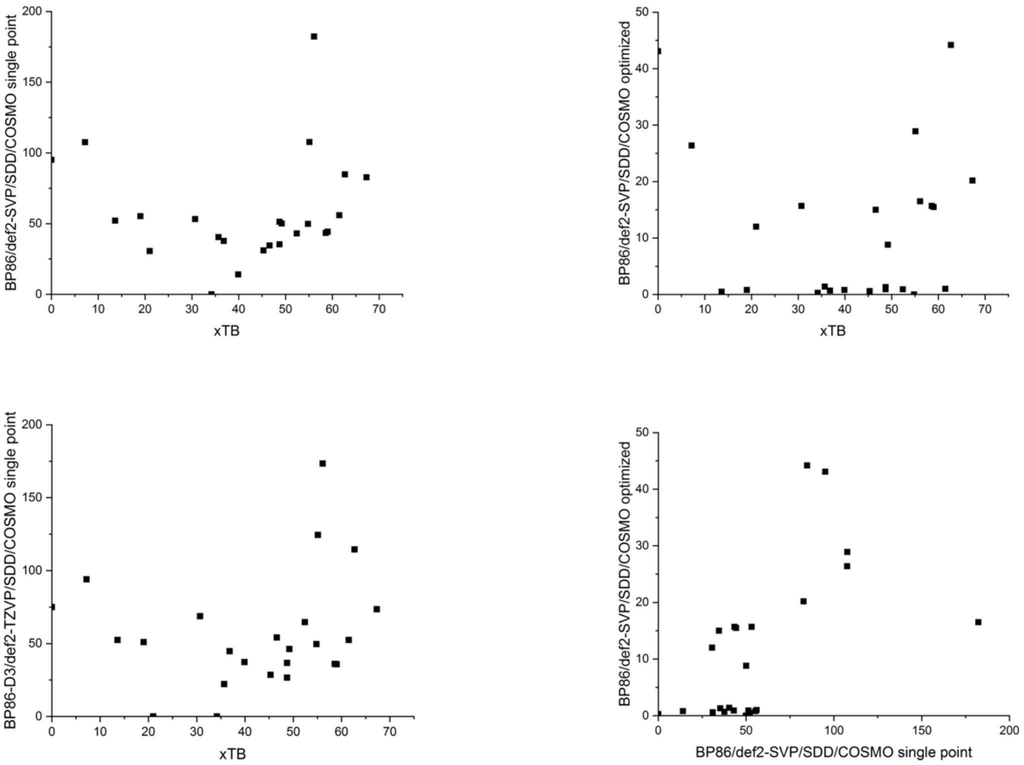

Figure S1. Energies of xTB optimized cluster representatives of cationic **product I** plotted against DFT single point energies (top and bottom left) and against DFT optimized energies (top right) as well as DFT single point energies on the xTB optimized clusters plotted against full DFT re-optimization (bottom right).

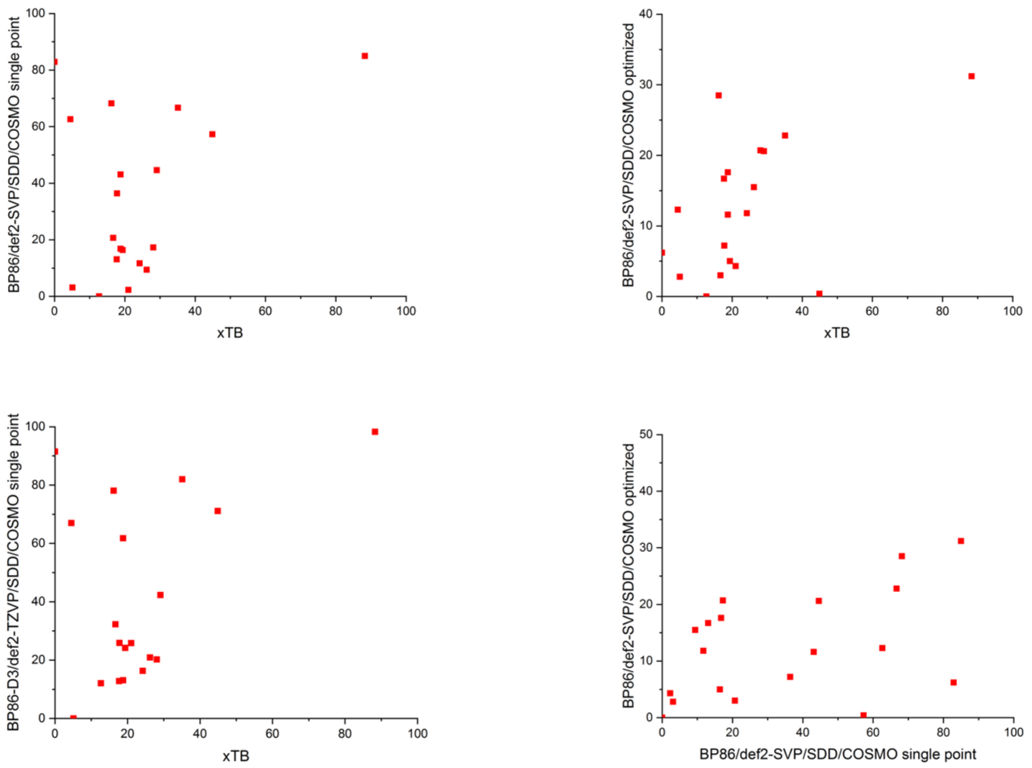

Figure S2. Energies of xTB optimized cluster representatives of cationic **product II** plotted against DFT single point energies (top and bottom left) and against DFT optimized energies (top right) as well as DFT single point energies on the xTB optimized clusters plotted against full DFT re-optimization (bottom right).

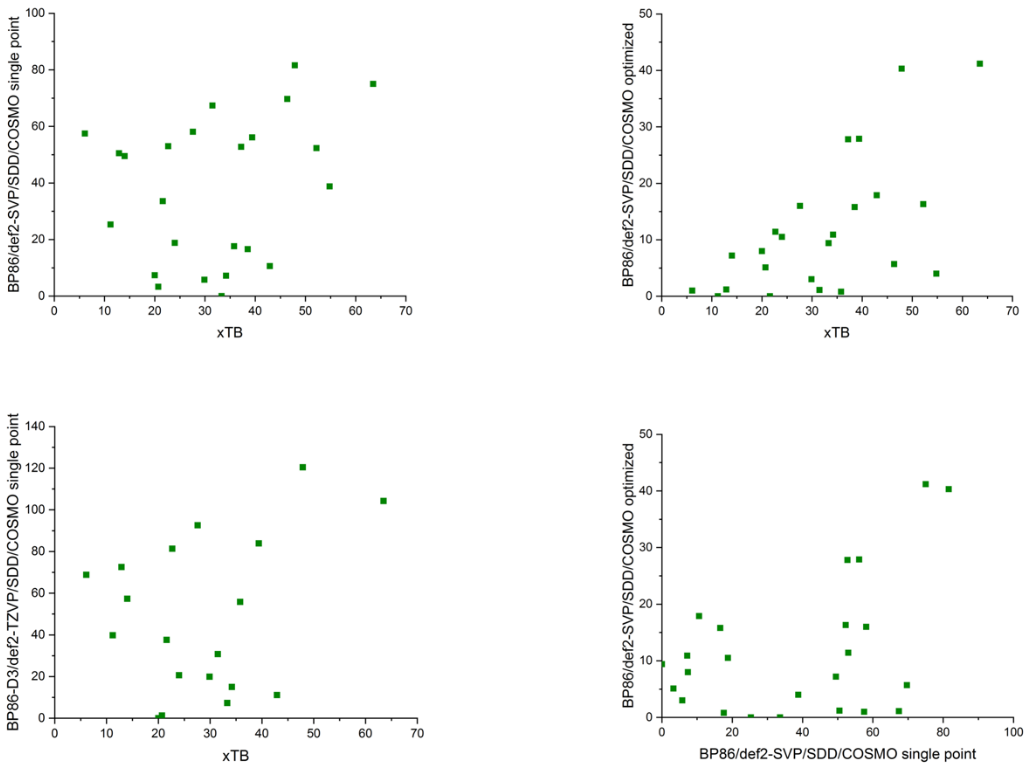

Figure S3. Energies of xTB optimized cluster representatives of cationic **product III** plotted against DFT single point energies (top and bottom left) and against DFT optimized energies (top right) as well as DFT single point energies on the xTB optimized clusters plotted against full DFT re-optimization (bottom right).

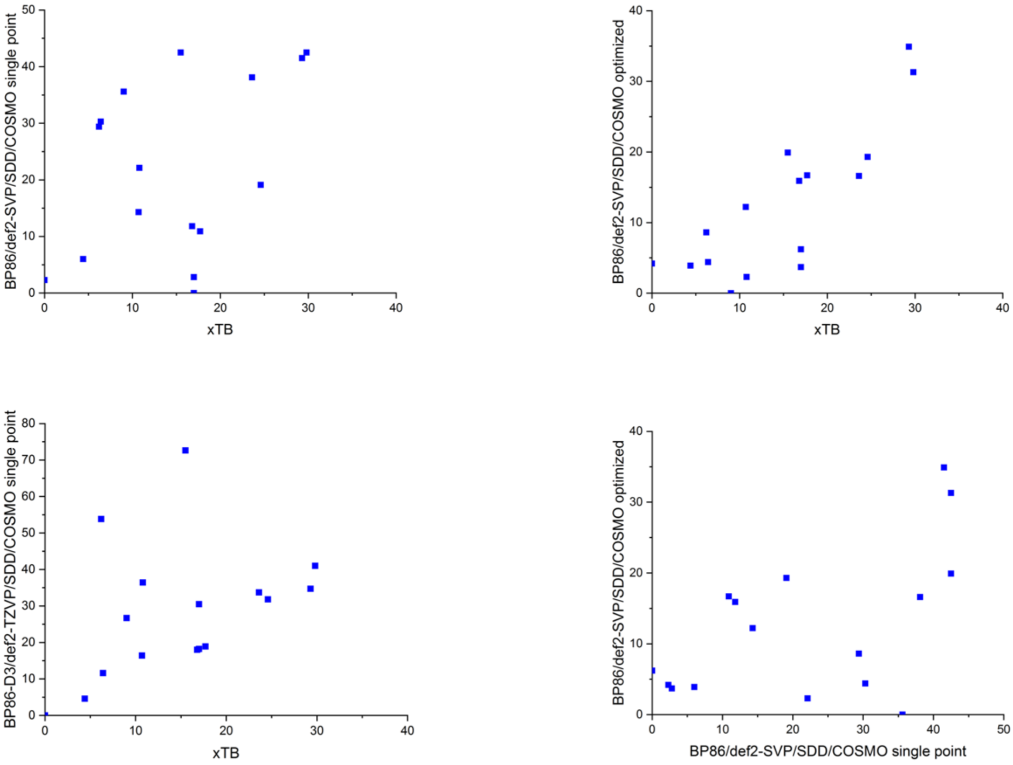

Figure S4. Energies of xTB optimized cluster representatives of **product IV** plotted against DFT single point energies (top and bottom left) and against DFT optimized energies (top right) as well as DFT single point energies on the xTB optimized clusters plotted against full DFT re-optimization (bottom right).

Figures S1 to S4 illustrate that the correlation between the relative energies of the xTB optimized cluster representatives and the relative energies obtained as DFT single points on the xTB optimized clusters is rather poor. Even when comparing DFT single point energies on obtained from the xTB optimized clusters with fully DFT optimized structures (bottom right graph in Figures S1 to S4), the correlation is still very weak. These findings prompted us to subject the representative cluster structures to full DFT re-optimization. As listed in Table S1 (last column), the outlined strategy was successful to identity conformers for each of the four stereoisomeric products with a lower energy than those obtained by chemical intuition, illustrating the power of automated approaches.

Non-Covalent Interaction

To reveal non-covalent interactions, the second eigenvalue of the electron-density Hessian matrix sign(λ_2)ρ is projected onto an isosurface of the reduced gradient *s*,[1] whereby a color-coding indicates the magnitude of sign(λ_2)ρ. Green areas characterize weak non-covalent interactions corresponding to a low (reduced) electron density gradient and a sign(λ_2)ρ close to zero. Blue areas characterize attractive interactions and corresponding to large negative values of sign(λ_2)ρ, whereas red areas indicate repulsive interactions corresponding to large positive values of sign(λ_2)ρ. All shown reduced gradient isosurfaces correspond to s = 0.4 a.u., whereas the values of sign(λ_2)ρ range from -0.1 a.u. (blue) to 0.1 a.u. (red). To calculate these properties the NCIPLOT tool[2] was used, while for the visualization VMD was employed.[3]

Comparison IMes vs IMesH$_2$

To further assess the effect of modifying the NHC from IMesH$_2$ to IMes, we performed a number of additional structure optimizations after modifying the corresponding fully characterized IMes species to IMesH$_2$. Minimum energy structures were fully optimized with BP86/def2-VP/SDD/COSMO, whereas energies of the transition states were only approximated. Here, the reaction coordinates were frozen, while all other parameters were allowed to fully relax. Energies of the IMesH$_2$ transition states are therefore upper bounds to the true transition states. As can be seen in Tables S2 and S3, energy differences for the fully optimized minimum energy species are very small.

Table S2. Comparison of relative electronic energies of various cationic intermediates and transition states of the investigated catalyst **1** with NHC=IMes and the original catalyst **1’** with NHC=IMesH_2. BP86/def2-SVP/SDD denotes energy obtained from full structure optimizations in implicit solvent, while BP86-D3/def2-TZVP/SDD denotes single point energies in implicit solvent as calculated on the BP86/def2-SVP/SDD optimized structures. Energies in kJ mol^{-1}.

	**1** (NHC=IMes)		**1’** (NHC=IMesH_2)	
	BP86/def2-SVP	BP86-D3/def2-TZVP	BP86/def2-SVP	BP86-D3/def2-TZVP
Adduct **I**	0.0	0.0	0.0	0.0
Adduct **II**	10.1	14.2	9.5	14.0
Adduct **III**	35.7	45.7	37.0	46.7
$TS_{cycloadd}$ **III**	54.3	68.8	54.6[a]	67.7[a]
Molybdacyclobutane **III**	-57.6	-44.6	-61.1	-49.4
$TS_{cyclorev.}$ **III**	-15.2	-12.5	-34.0[a]	-23.1[a]
Product **III**	-101.7	-27.1	-101.7	-21.2

[a] Upper bounds.

Table S3. Comparison of relative electronic energies of starting material, intermediates and transition states of the investigated catalyst **1** with NHC=IMes and the original catalyst **1'** with NHC=IMesH_2. In all instances a neutral species is considered, either with a bound triflate (Cat$_{syn}$ and Cat$_{anti}$) or as an ion complex with a dissociated triflate. BP86/def2-SVP/SDD denotes energy obtained from full structure optimizations in implicit solvent, while BP86-D3/def2-TZVP/SDD denotes single point energies in implicit solvent as calculated on the BP86/def2-SVP/SDD optimized structures. Energies in kJ mol^{-1}.

	**1** (NHC=IMes)		**1'** (NHC=IMesH_2)	
	BP86/def2-SVP	BP86-D3/def2-TZVP	BP86/def2-SVP	BP86-D3/def2-TZVP
Cat$_{syn}$+NBE	0.0	0.0	0.0	0.0
Cat$_{anti}$+NBE	18.6	10.2	18.8	15.7
TS$_{associative}$ (**IIIb**)	154.5	63.8	161.9[a]	73.0[a]
TS$_{SN2}$ (**IIIc**)	157.3	68.2	165.2[a]	79.6[a]
Molybdacyclobutane III with OTf$^-$	54.2	-47.8	47.6	-40.5

[a] Upper bounds.

Reaction Energy Profiles

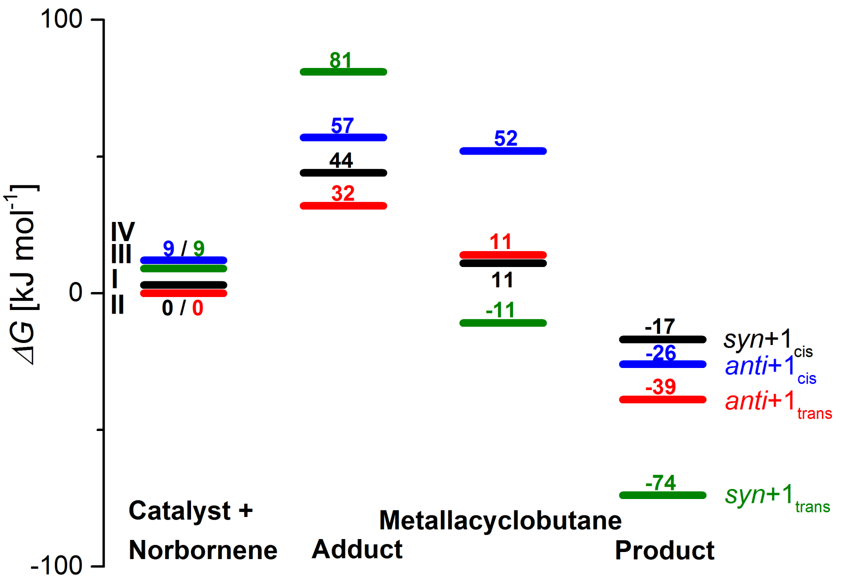

Figure S5. Relative free energy of reaction intermediates in which the cationic species (adduct, molybdacyclobutane, product) each forms an ion pair with the dissociated triflate anion. Reaction free energies are calculated with BP86/def2-TZVP/D3/COSMO//BP86/def2-SVP/COSMO at 303 K, where the solvent is modelled with a dielectric constant of $\varepsilon = 9$ (see also Computational Methodology).

As visible in Figure S5, the anion exerts a stabilizing effect on the complexes. An earlier study showed that this stabilizing effect is roughly the same for all species.[4] Yet, it seems that the most stable product (green) benefits more from the complexation with triflate than the other product isomers. Infact, in all cationic insertion products recoordination of the dissociated triflate resulted in more stable structures than the formation of an ion complex of the cationic product and the anionic triflate. However, this finding should not be overinterpreted due to the inherent difficulty to adequately sample the triflate positions to find the most stable arrangement. In addition, it is likely that this stabilizing effect is to some extend quenched by the shielding effects of surrounding solvent molecules.

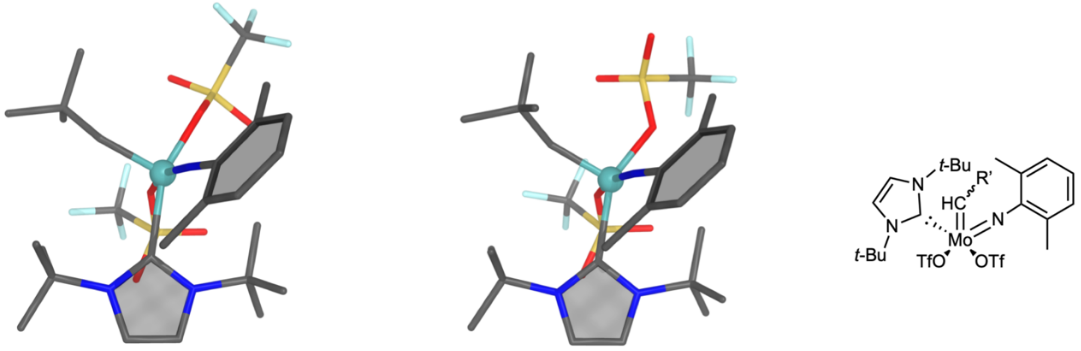

Figure S6. Molecular structure of catalyst **2** (Mo(N-2,6-Me_2-C_6H_3)($CHCMe_3$)(1,3-di-*t*-butyl-1,3-dihydro-2H-imidazol-2-ylidene)($OTf)_2$) in its *anti*- (left) and *syn*-conformation (middle), Lewis formula (right).

We also investigated a second related catalysts **2** with a different *N*-heteocyclic carbene (Mo(N-2,6-Me_2-C_6H_3)($CHCMe_3$)(I-*t*Bu)($OTf)_2$ with I-*t*Bu = 1,3-di-*t*-butyl-1,3-dihydro-2H-imidazol-2-ylidene). This species was found to yield lower *trans* content in the polymer. In the crystal structure the catalyst is in its *syn* conformation.[5] The relative free energy difference between the *syn*- and the *anti*-conformation of **2** is 31.7 kJ mol^{-1} and about three times larger than for **1**.

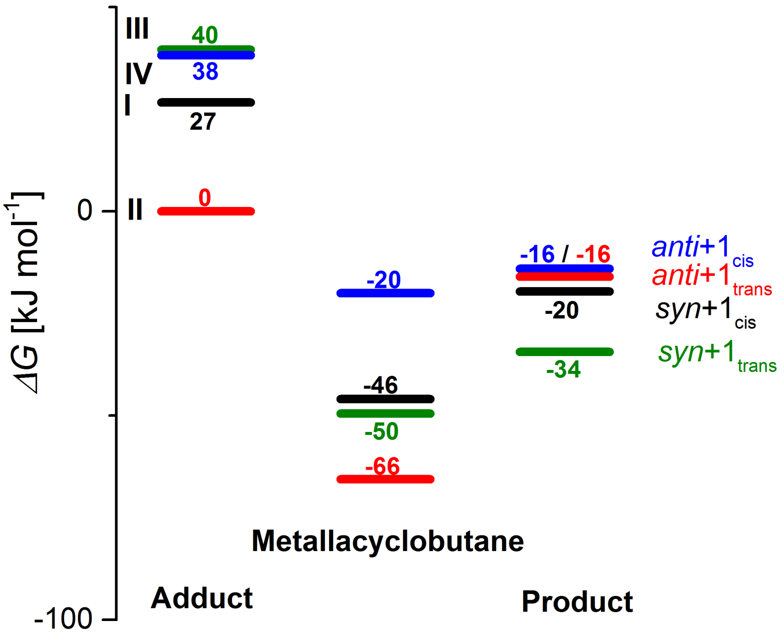

Figure S7. Relative reaction free energy of the cationic adducts, molybdacyclobutane, and product of the various stereoisomers of **2** when reacting with norbornene. Reaction free energies are obtained with BP86/def2-TZVP/D3/COSMO//BP86/def2-SVP/COSMO at 303 K, where the solvent is modelled with a dielectric constant of $\varepsilon = 9$ (see also Computational Methodology).

Visualization of Non-Covalent Interactions in Molybdacyclobutane Intermediates

Figures S8 to S13 depict the non-covalent interactions in the molybdacyclobutane **I** to **IV** of **1** and molybdacyclobutane **I** and **II** of **2**. Next to the visualization of these interactions based on the analysis of the reduced gradient and Hessian, schematic representations are depicted to decipher the complex intramolecular interactions.

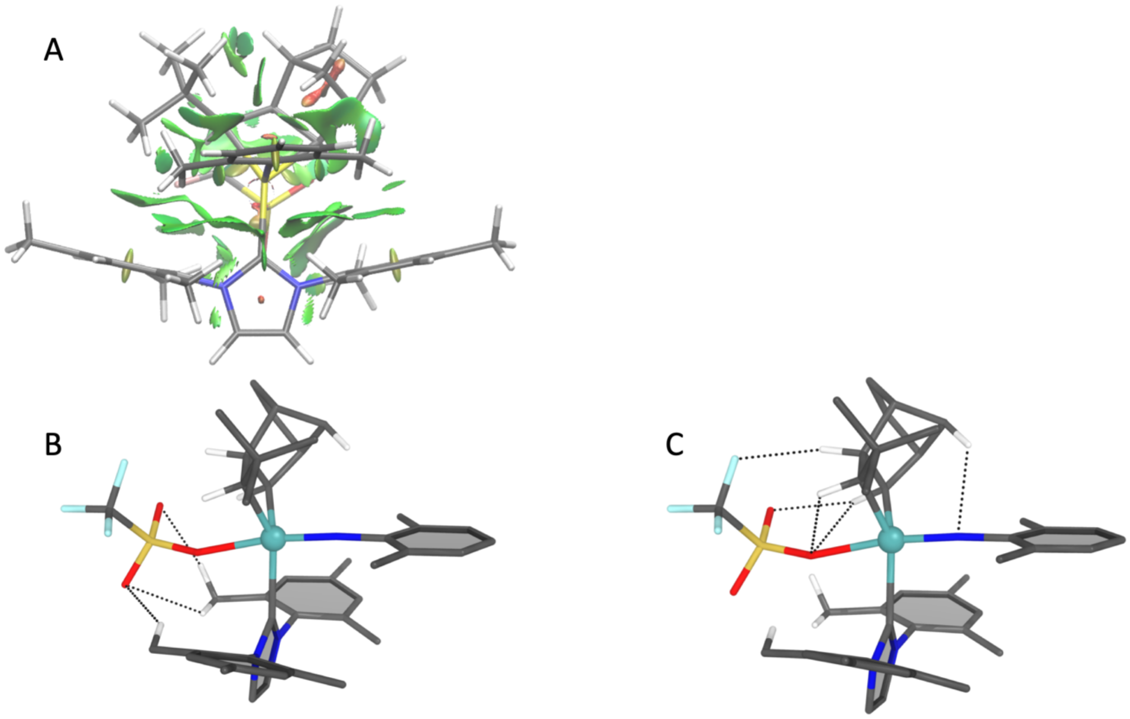

Figure S8. Visualization of non-covalent interactions in the **metallacyclobutane I** of **1**.A. Isosurface of the reduced gradient (*s* = 0.4 a.u.), where blue areas indicate strongly attractive interactions, green weakly attractive van der Waals like interactions, and red strongly repulsive interaction. B. Schematic representation of the interactions between the NHC and the triflate. C. Schematic interactions between the metallacyclobutane and alkylidene unit with the triflate and the imido nitrogen.

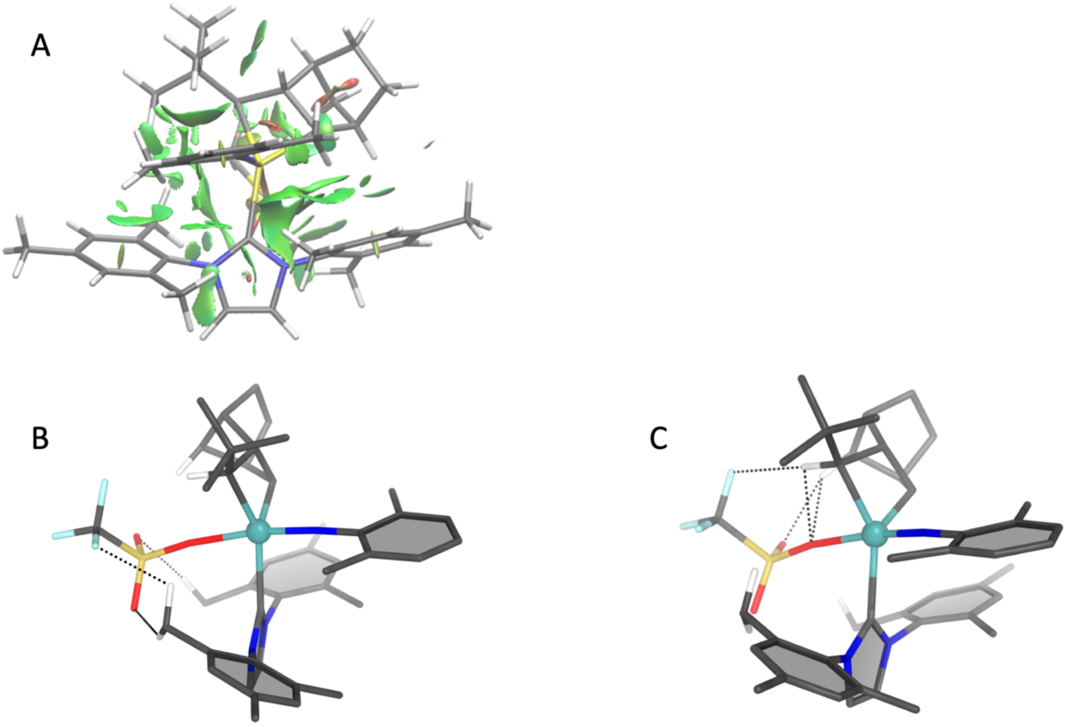

Figure S9. Visualization of non-covalent interactions in the **metallacyclobutane II** of **1**.A. Isosurface of the reduced gradient (*s* = 0.4 a.u.), where blue areas indicate strongly attractive interactions, green weakly attractive van der Waals like interactions, and red strongly repulsive interaction. B. Schematic representation of the interactions between the NHC and the triflate. C. Schematic interactions between the metallacyclobutane and the triflate.

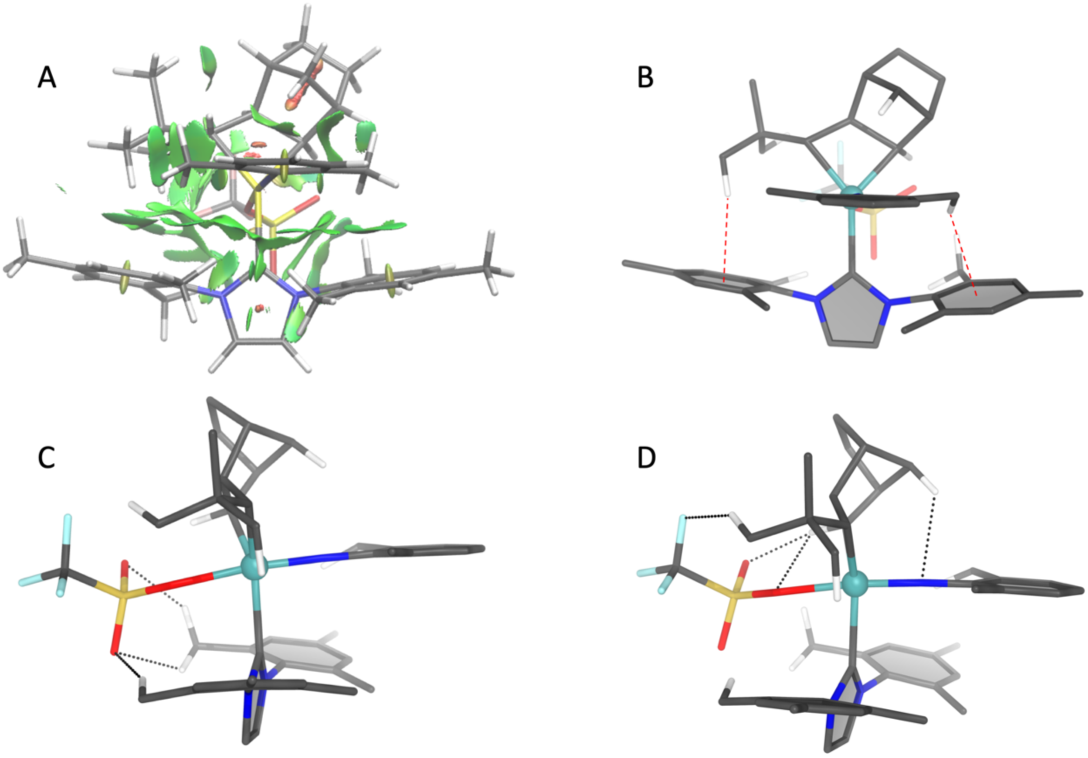

Figure S10. Visualization of non-covalent interactions in the **metallacyclobutane III** of **1**. A. Isosurface of the reduced gradient (*s* = 0.4 a.u.), where blue areas indicate strongly attractive interactions, green weakly attractive van der Waals like interactions, and red strongly repulsive interaction. B. Schematic representation of the non-covalent CH-π interactions between the NHC and the alkylidene and imido group with distances of 2.65 and 2.76 Å, respectively. C. Schematic representation of the interactions between NHC the triflate. D. Schematic representation of the interactions between the metallacyclobutane, the imido and the triflate.

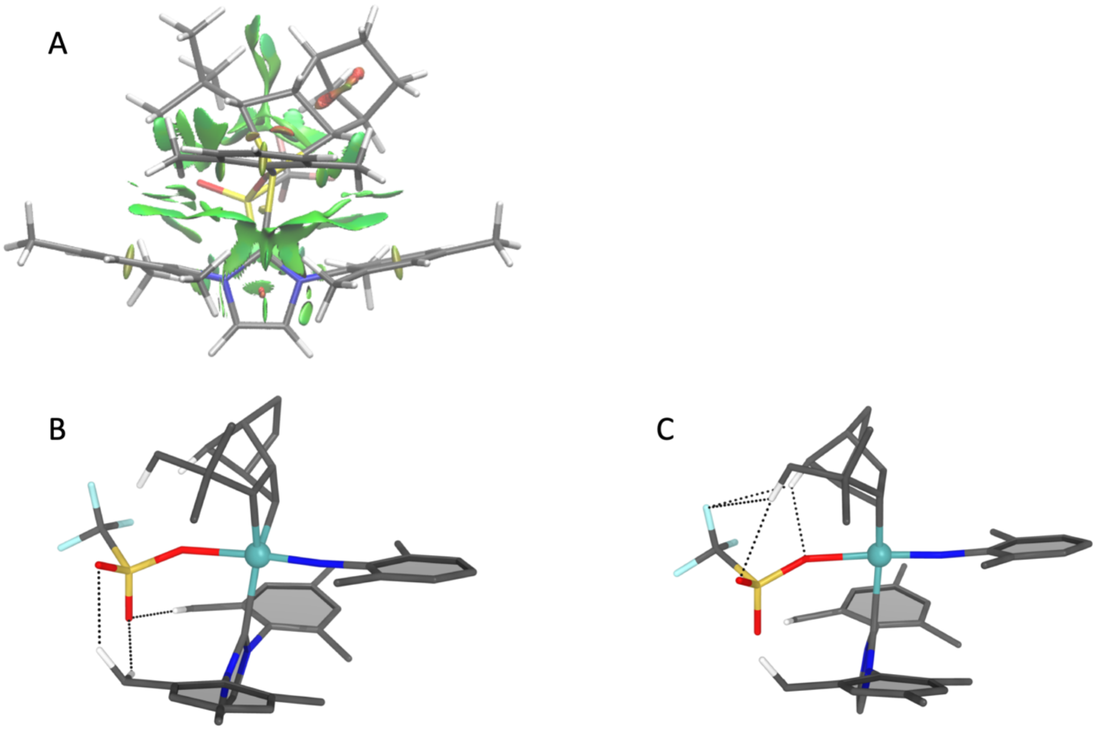

Figure S11. Visualization of non-covalent interactions in the **metallacyclobutane IV** of **1**. A. Isosurface of the reduced gradient (*s* = 0.4 a.u.), where blue areas indicate strongly attractive interactions, green weakly attractive van der Waals like interactions, and red strongly repulsive interaction. B. Schematic representation of the interactions between the NHC and the triflate. C. Schematic representation of the interactions between the metallacyclobutane, the alkylidene and the triflate.

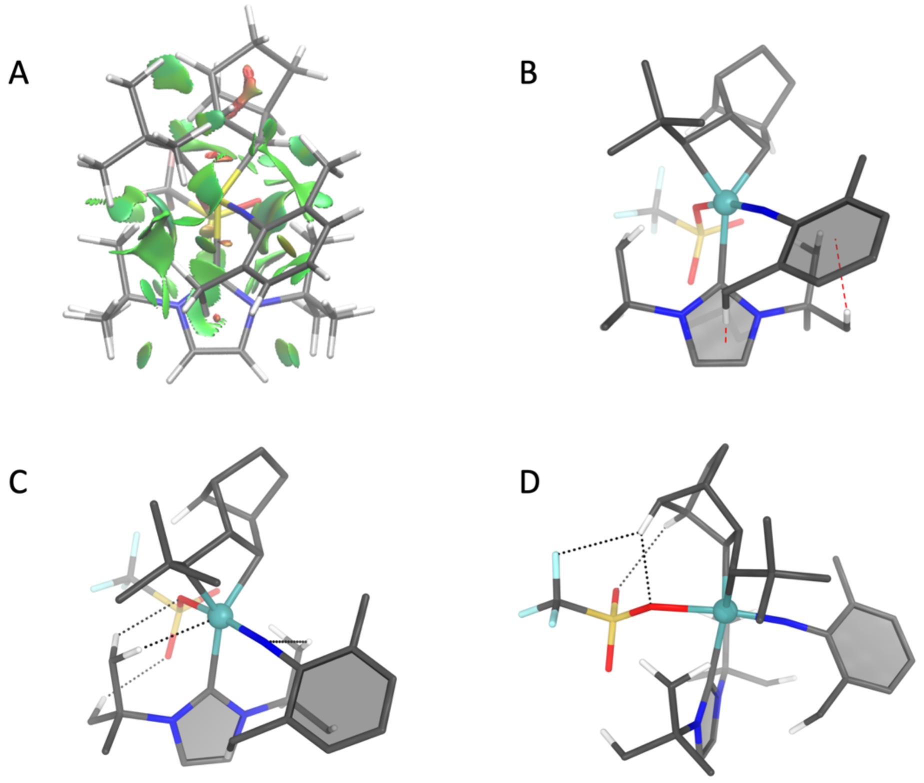

Figure S12. Visualization of non-covalent interactions in the **metallacyclobutane II** of **2**. A. Isosurface of the reduced gradient (s = 0.4 a.u.), where blue areas indicate strongly attractive interactions, green weakly attractive van der Waals like interactions, and red strongly repulsive interaction. B. Schematic representation of the CH-π interactions, with distances of 2.68 and 3.27 Å. C. Schematic representation of the interactions between the NHC and the remainder of the catalyst. D. Schematic representation of the interactions between the metallacyclobutane and the triflate.

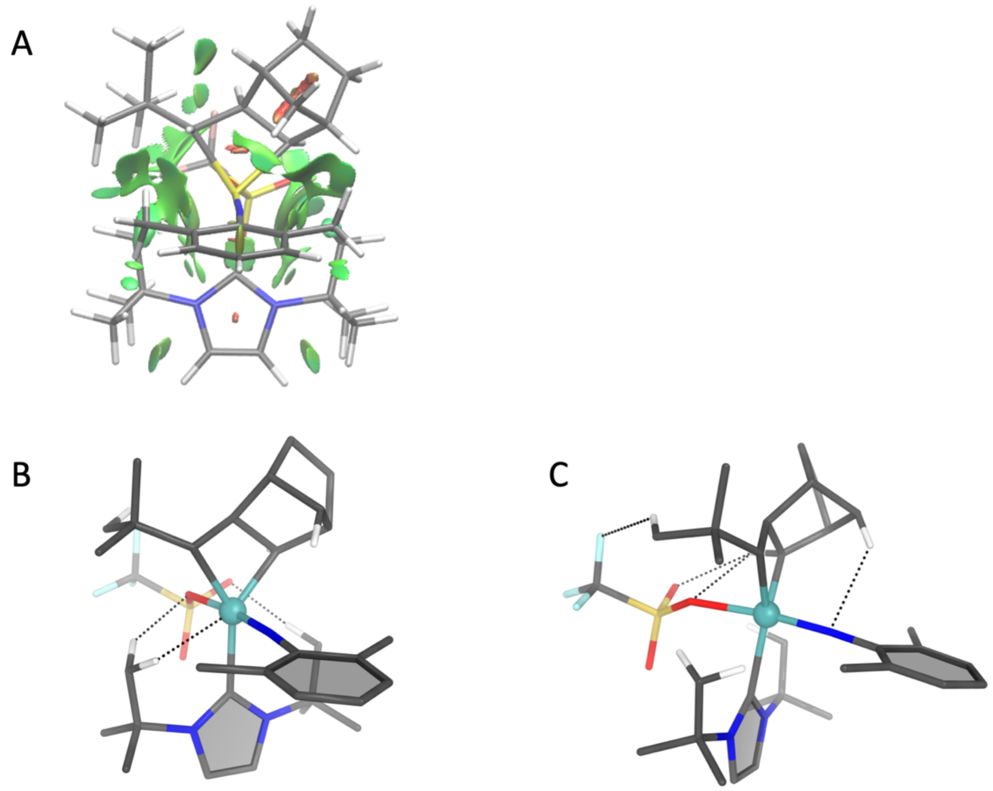

Figure S13. Visualization of non-covalent interactions of **metallacyclobutane III** of **2**. A. Isosurface of the reduced gradient (*s* = 0.4 a.u.), where blue areas indicate strongly attractive interactions, green weakly attractive van der Waals like interactions, and red strongly repulsive interaction. B. Schematic representation between the interactions of the NHC and the triflate. C. Schematic representation of the intramolecular interactions of the metallacyclobutane and alkylidene with the rest of the catalyst.

Structures of the Investigated Species

In the following, the 3D structures of the quantum chemically optimized species, catalyst, intermediates, transition states, and the products of **1** are depicted in Figures S14-S41, whereas those of **2** are depicted in Figures S42-S55. Conformer searches were performed for all stereoisomeric products of **1** and **2** and selected other intermediates as indicated in the figure captions. As the four products of **2** were derived from the most stable conformers of the respective structures of **1**, conformer searches identified energetically more favorable species only for the *anti*+1_{cis} (product II) and the *anti*+1_{trans} (product IV) species. Cartesian coordinates of each species are listed in a separate file.

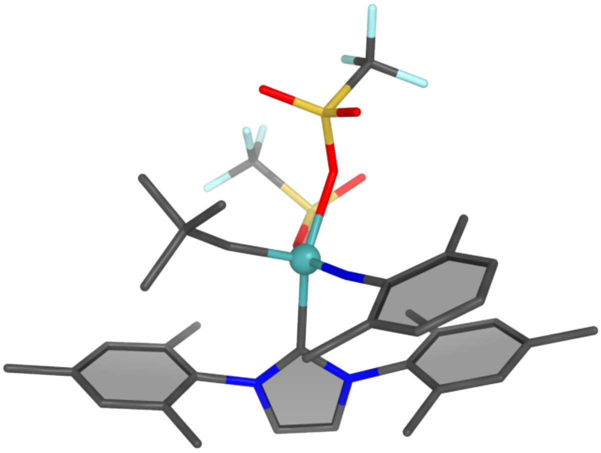

Figure S14. BP86/def2-SVP/COSMO (ε = 9.0) optimized structure, *syn*-**1**.

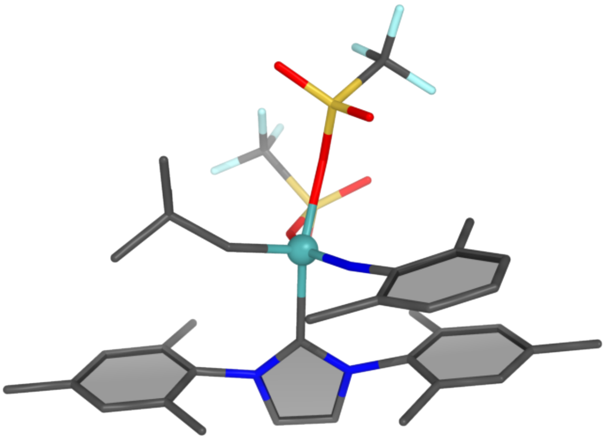

Figure S15. BP86/def2-SVP/COSMO (ε = 9.0) optimized structure, *anti*-**1**.

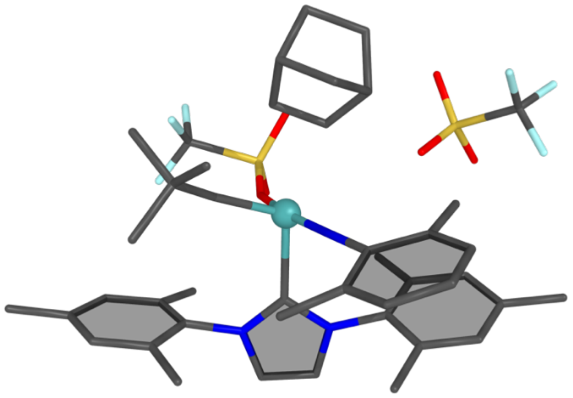

Figure S16. BP86/def2-SVP/COSMO (ε = 9.0) optimized structure of **adduct I**, ion pair with dissociated triflate.

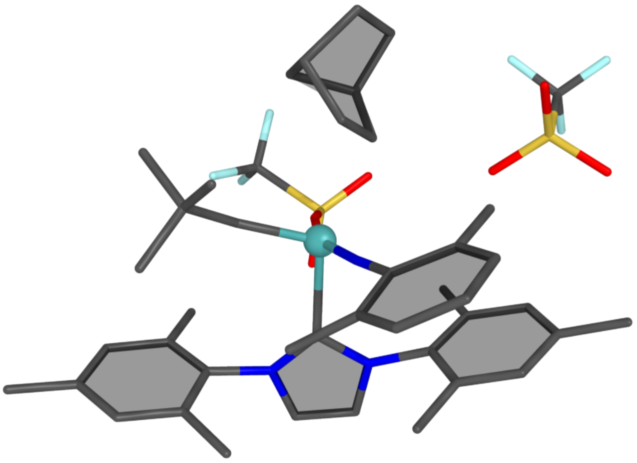

Figure S17. BP86/def2-SVP/COSMO (ε = 9.0) optimized structure of the **adduct II**, ion pair with dissociated triflate.

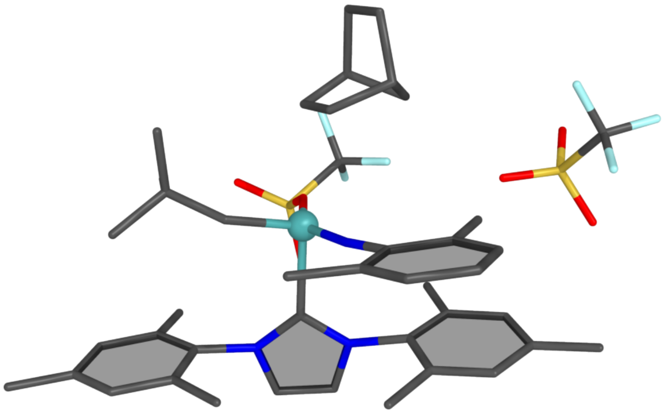

Figure S18. BP86/def2-SVP/COSMO (ε = 9.0) optimized structure of the **adduct III**, ion pair with dissociated triflate.

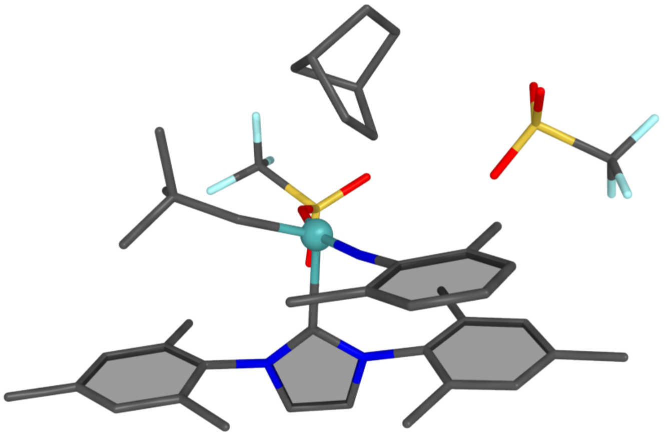

Figure S19. BP86/def2-SVP/COSMO (ε = 9.0) optimized structure of the **adduct IV**, ion pair with dissociated triflate.

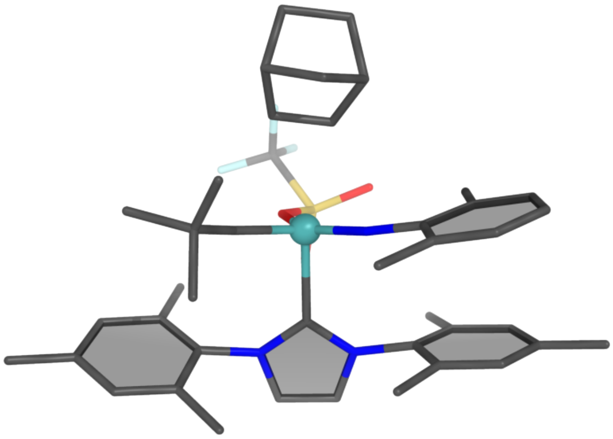

Figure S20. BP86/def2-SVP/COSMO (ε = 9.0) optimized structure of the **cationic adduct I**.

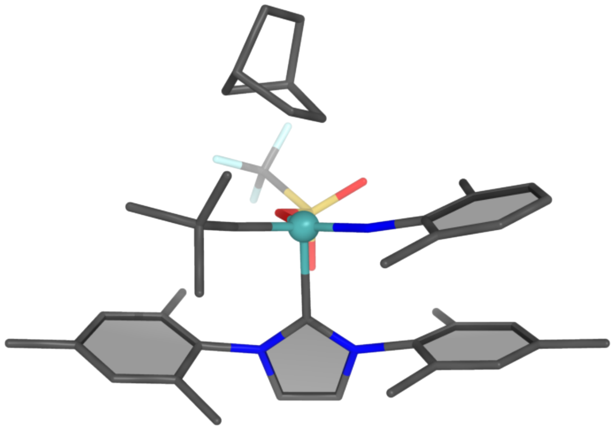

Figure S21. BP86/def2-SVP/COSMO (ε = 9.0) optimized structure of the **cationic adduct II**.

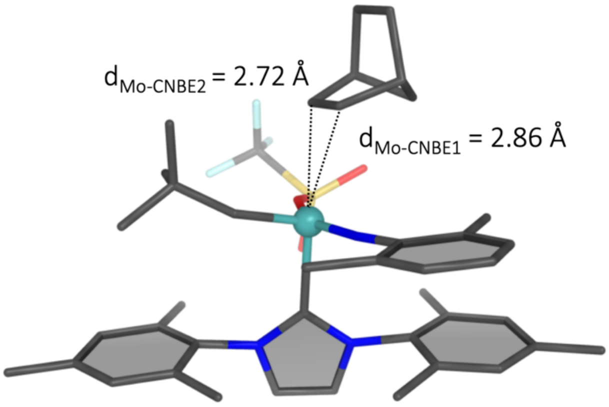

Figure S22. BP86/def2-SVP/COSMO (ε = 9.0) optimized structure of the **cationic adduct III**.

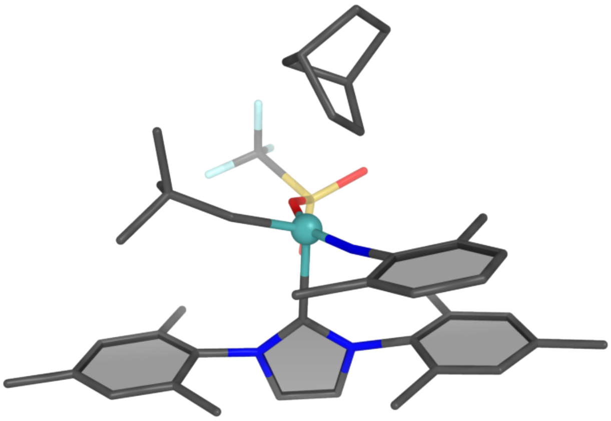

Figure S23. BP86/def2-SVP/COSMO (ε = 9.0) optimized structure of the **cationic adduct VI**.

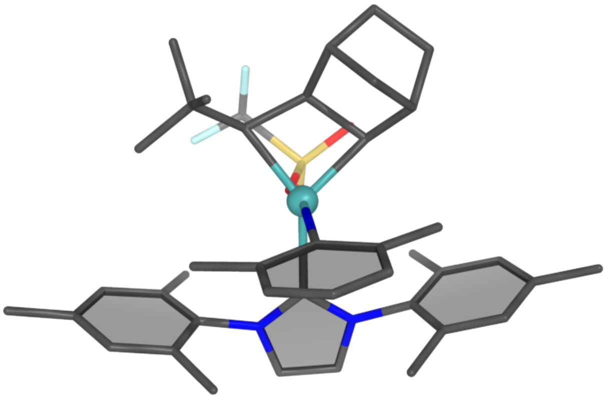

Figure S24. BP86/def2-SVP/COSMO (ε = 9.0) optimized structure of the **metallacyclobutane I**.

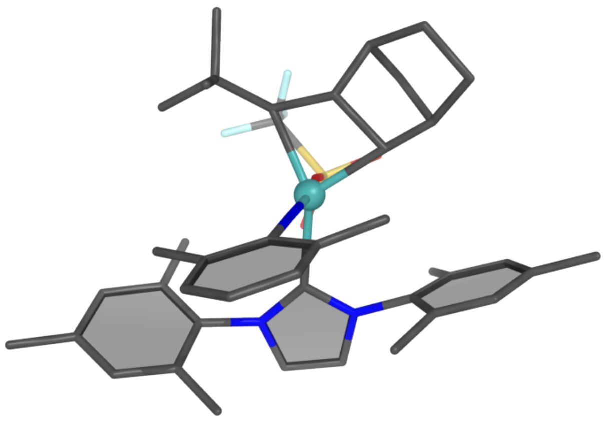

Figure S25. BP86/def2-SVP/COSMO ($\varepsilon = 9.0$) optimized structure of the **metallacyclobutane II**.

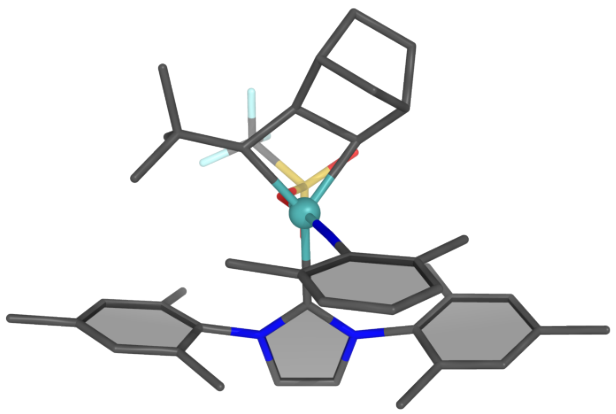

Figure S26. BP86/def2-SVP/COSMO ($\varepsilon = 9.0$) optimized structure of the **metallacyclobutane III**.

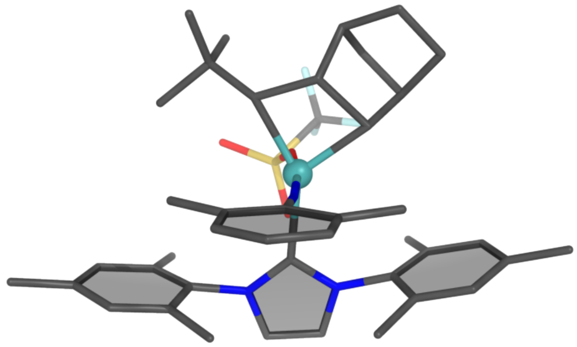

Figure S27. BP86/def2-SVP/COSMO ($\varepsilon = 9.0$) optimized structure of the **metallacyclobutane VI**. The most stable conformation was identified after extensive conformer search (see Computational Methodology for details).

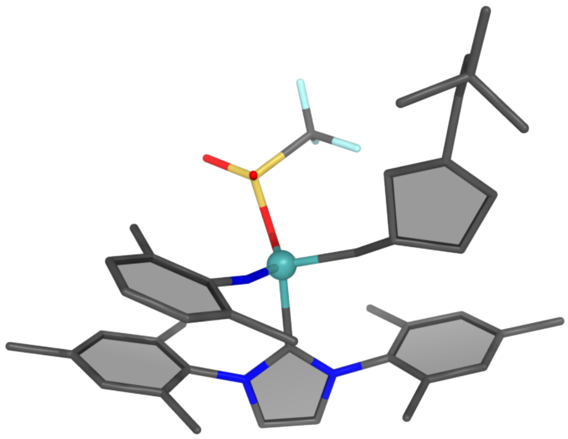

Figure S28. BP86/def2-SVP/COSMO ($\varepsilon = 9.0$) optimized structure of the *syn*+1_{cis} conformer (**product I**). The most stable conformation was identified after extensive conformer search (see Computational Methodology for details).

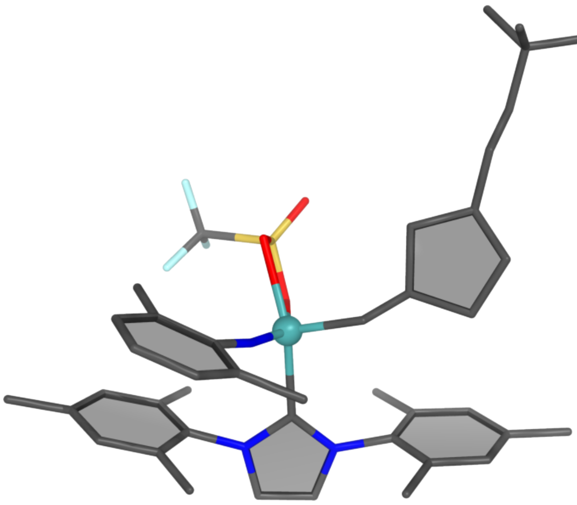

Figure S29. BP86/def2-SVP/COSMO ($\varepsilon = 9.0$) optimized structure of the *anti*+1_{trans} conformer (**product II**). The most stable conformation was identified after extensive conformer search (see Computational Methodology for details).

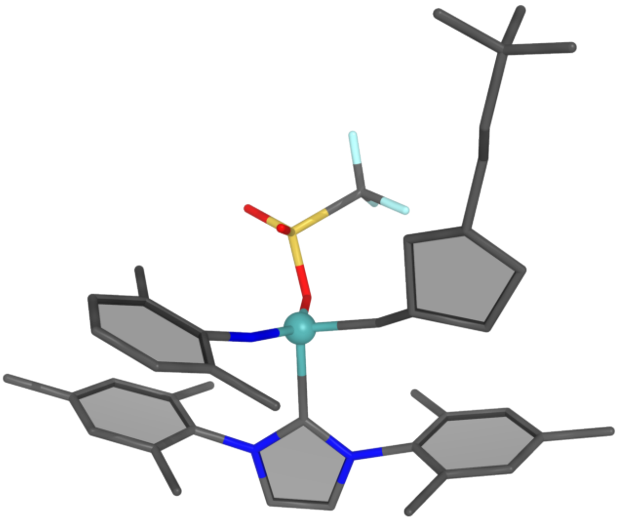

Figure S30. BP86/def2-SVP/COSMO (ε = 9.0) optimized structure of the *syn*+1_{trans} conformer (**product III**). The most stable conformation was identified after extensive conformer search (see Computational Methodology for details).

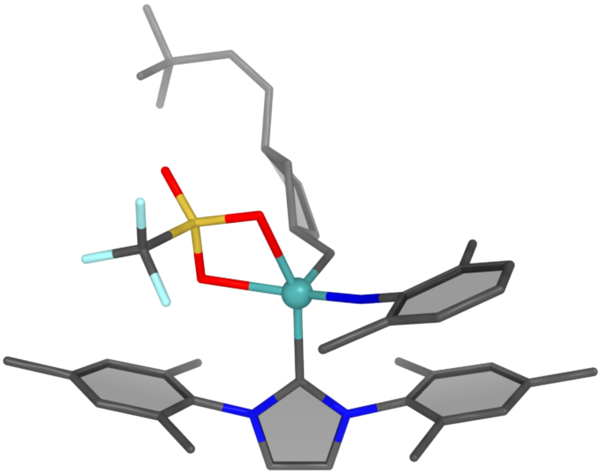

Figure S31. BP86/def2-SVP/COSMO (ε = 9.0) optimized structure of the *anti*+1_{cis} conformer (**product IV**). The most stable conformation was identified after extensive conformer search (see Computational Methodology for details).

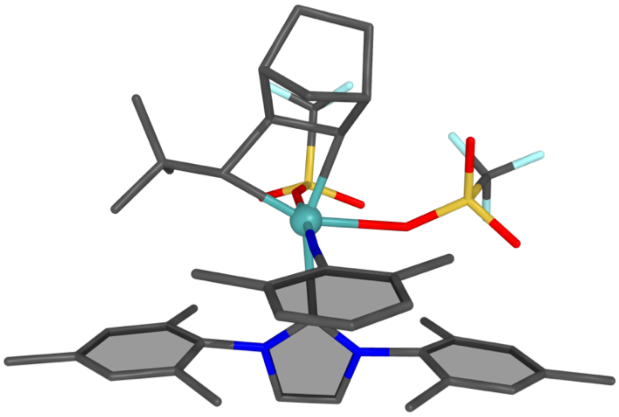

Figure S32. BP86/def2-SVP/COSMO (ε = 9.0) optimized structure of the **metallacyclobutane III** with bound triflate.

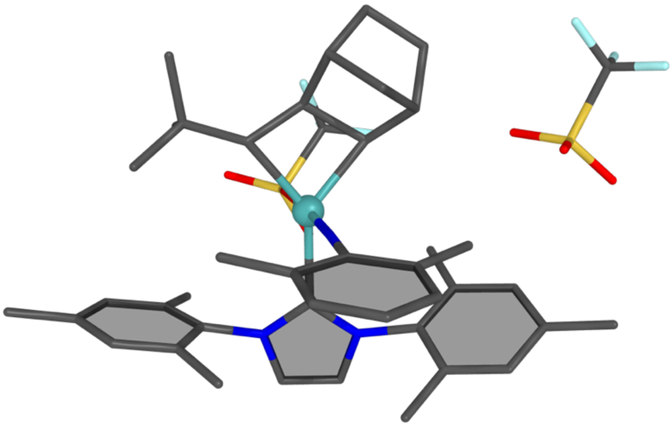

Figure S33 BP86/def2-SVP/COSMO (ε = 9.0) optimized structure of the **metallacyclobutane III**, ion pair with dissociated triflate.

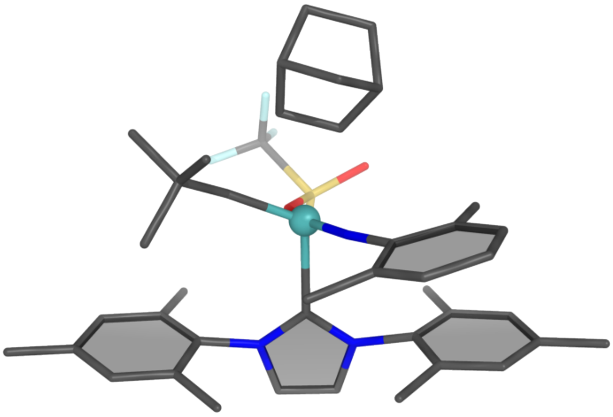

Figure S34. BP86/def2-SVP/COSMO (ε = 9.0) optimized structure of $\mathbf{TS_{Cycloadd.}}$ of **I**.

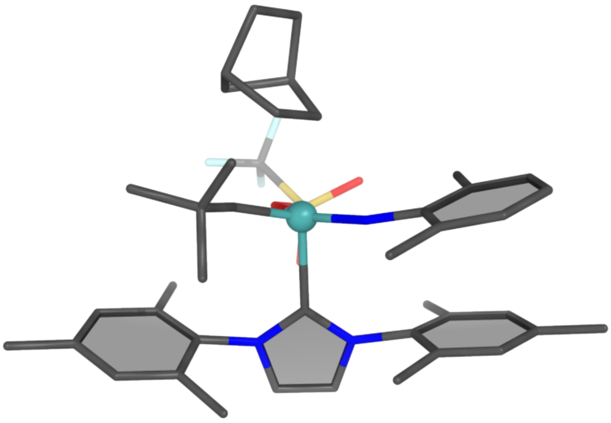

Figure S35. BP86/def2-SVP/COSMO (ε = 9.0) optimized structure of $\mathbf{TS_{Cycloadd.}}$ of **II**.

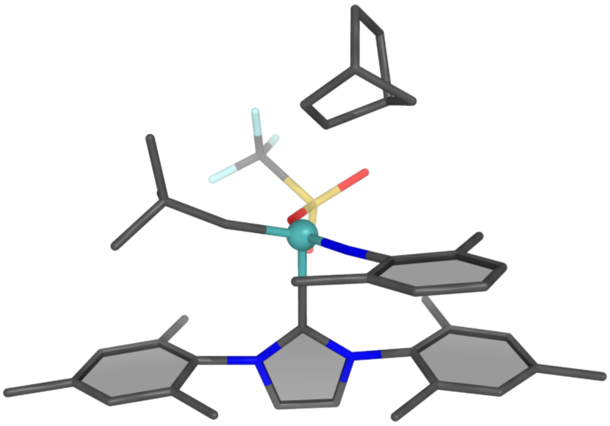

Figure S36. BP86/def2-SVP/COSMO (ε = 9.0) optimized structure of $\mathbf{TS_{Cycloadd.}}$ of **III**.

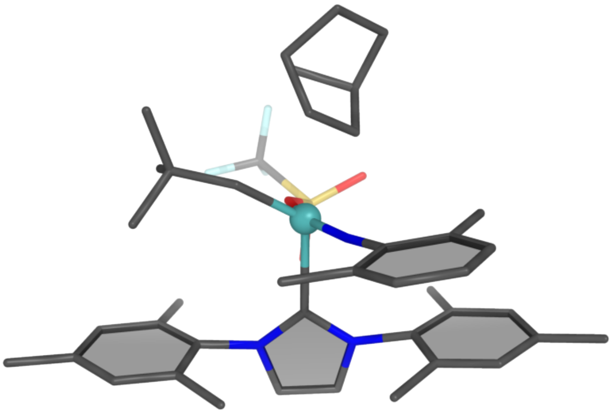

Figure S37. BP86/def2-SVP/COSMO (ε = 9.0) optimized structure of $\mathbf{TS_{Cycloadd.}}$ of **IV**.

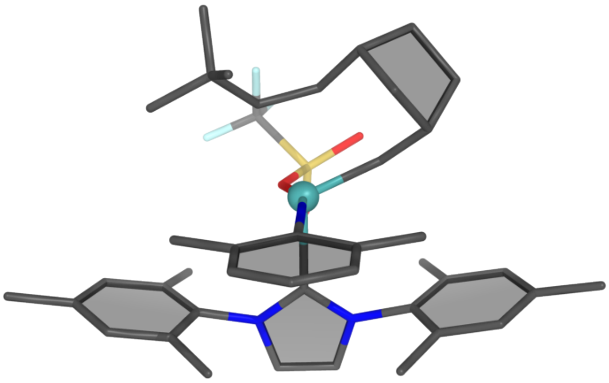

Figure S38. BP86/def2-SVP/COSMO (ε = 9.0) optimized structure of $\mathbf{TS}_{\mathbf{Cyclorev.}}$ of **I**.

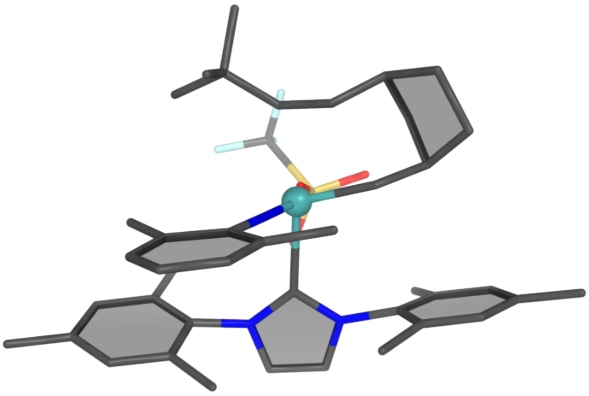

Figure S39. BP86/def2-SVP/COSMO (ε = 9.0) optimized structure of $\mathbf{TS}_{\mathbf{Cyclorev.}}$ of **II**.

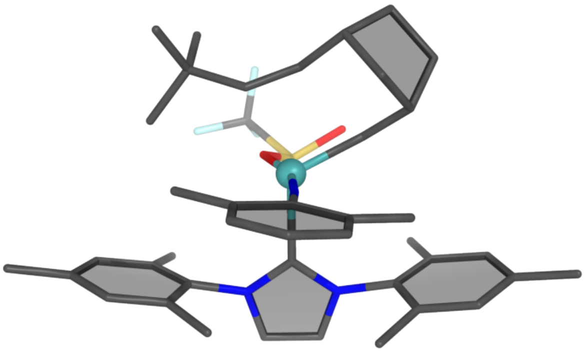

Figure S40. BP86/def2-SVP/COSMO (ε = 9.0) optimized structure of $\mathbf{TS}_{\mathbf{Cyclorev.}}$ of **III**.

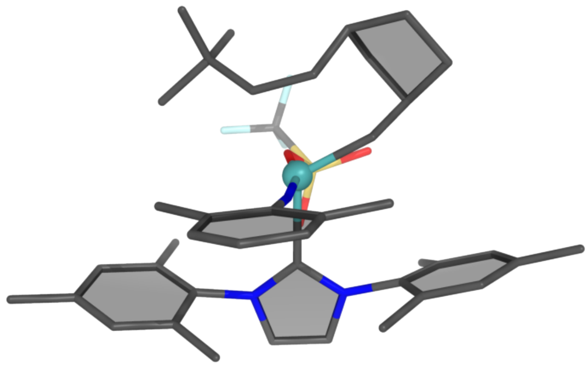

Figure S41. BP86/def2-SVP/COSMO (ε = 9.0) optimized structure of **TS**$_{\mathbf{Cyclorev.}}$ of **IV**.

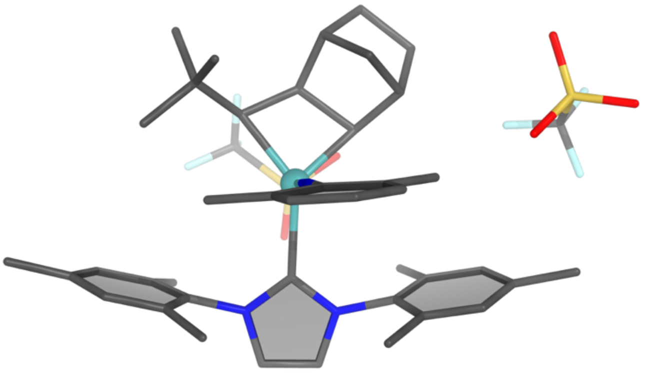

Figure S42. BP86/def2-SVP/COSMO (ε = 9.0) optimized structure of **molybdacyclobutane I** in complex with OTf$^-$.

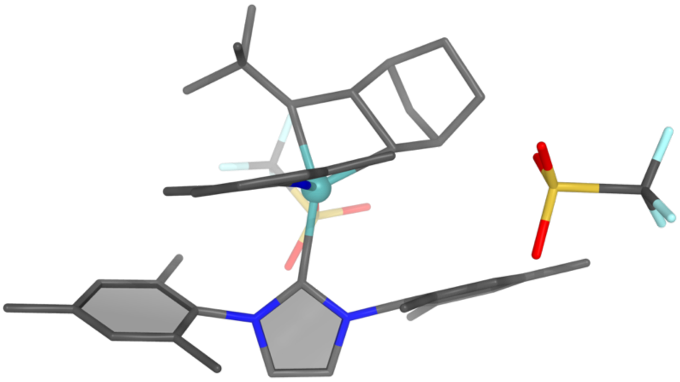

Figure S43. BP86/def2-SVP/COSMO (ε = 9.0) optimized structure of **molybdacyclobutane II** in complex with OTf$^-$.

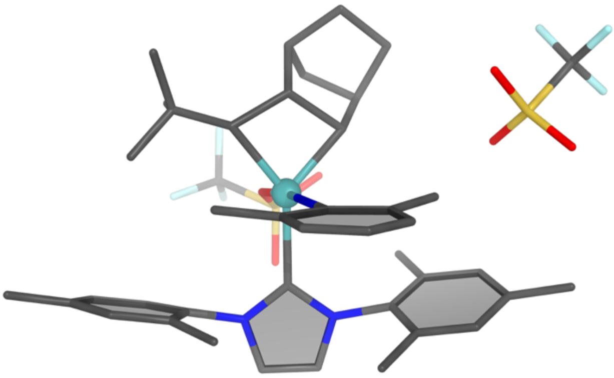

Figure S44. BP86/def2-SVP/COSMO (ε = 9.0) optimized structure of **molybdacyclobutane IV** in complex with OTf^-.

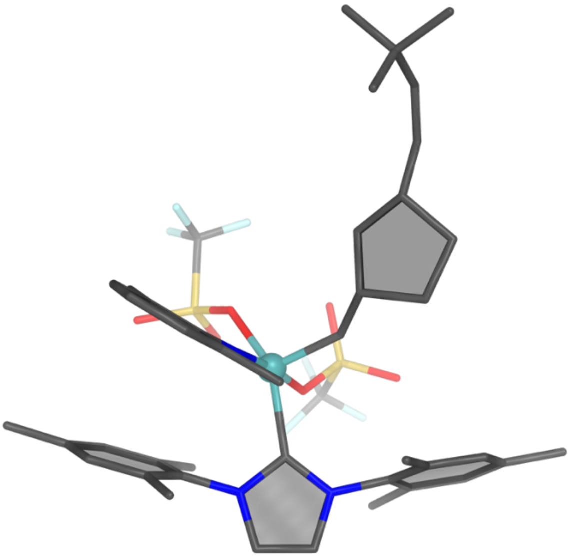

Figure S45. BP86/def2-SVP/COSMO (ε = 9.0) optimized structure of **product I** in complex with OTf^-.

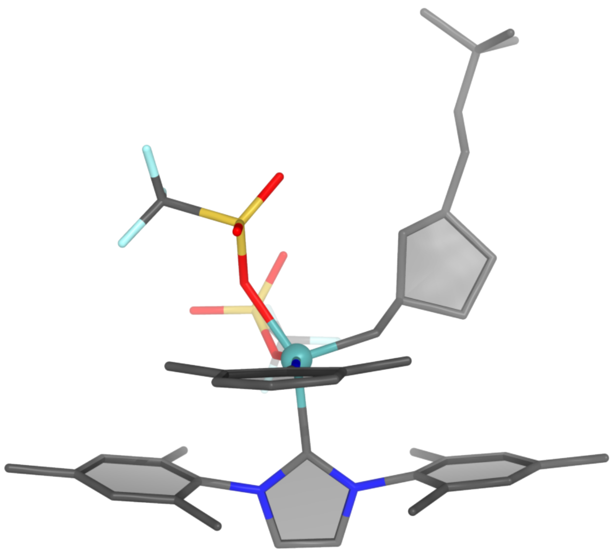

Figure S46. BP86/def2-SVP/COSMO ($\varepsilon = 9.0$) optimized structure of **product II** with coordinated OTf^-.

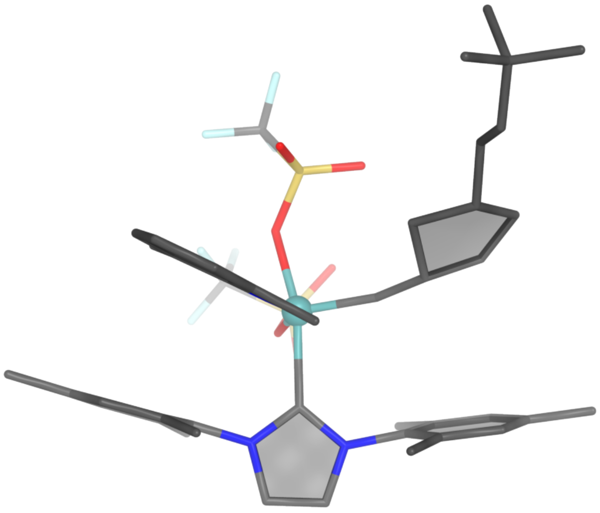

Figure S47. BP86/def2-SVP/COSMO ($\varepsilon = 9.0$) optimized structure of **product III** with coordinated OTf^-.

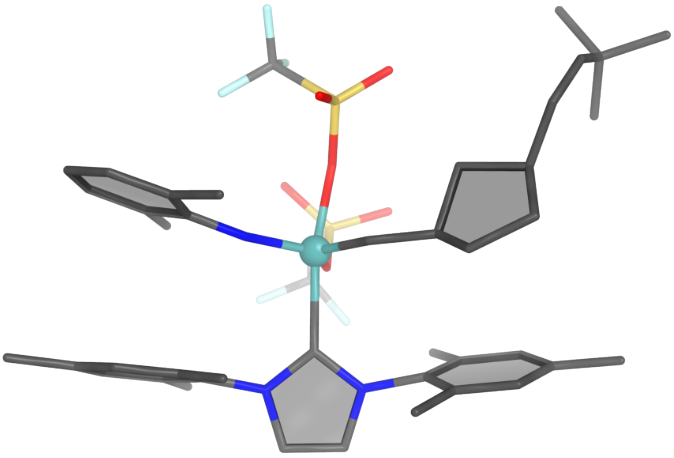

Figure S48. BP86/def2-SVP/COSMO (ε = 9.0) optimized structure of **product IV** with coordinated OTf^-.

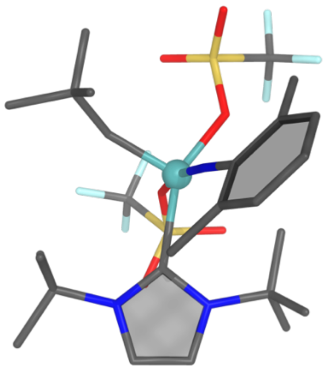

Figure S49. BP86/def2-SVP/COSMO (ε = 9.0) optimized structure of *syn*-**2**.

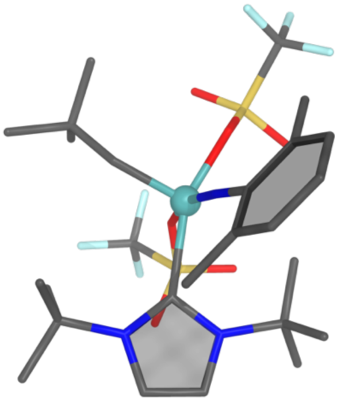

Figure S50. BP86/def2-SVP/COSMO (ε = 9.0) optimized structure of *anti*-**2**.

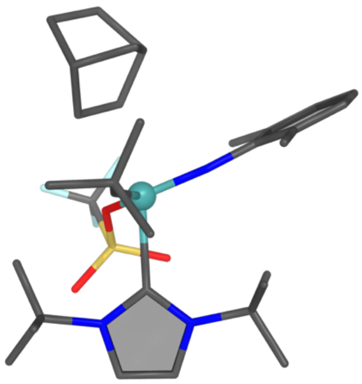

Figure S51. BP86/def2-SVP/COSMO (ε = 9.0) optimized cationic **adduct I** structure of **2**.

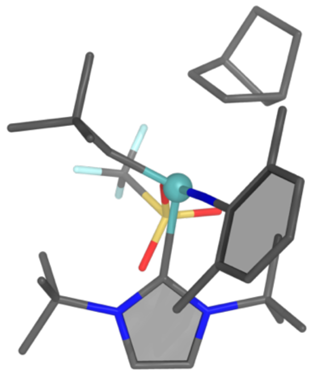

Figure S52. BP86/def2-SVP/COSMO (ε = 9.0) optimized cationic **adduct II** structure of **2**.

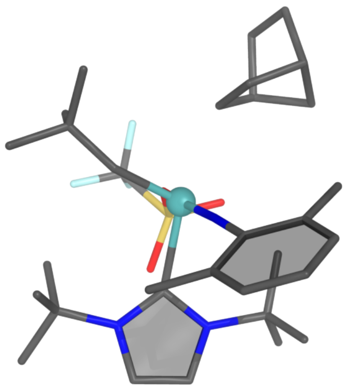

Figure S53. BP86/def2-SVP/COSMO (ε = 9.0) optimized cationic **adduct III** structure of **2**.

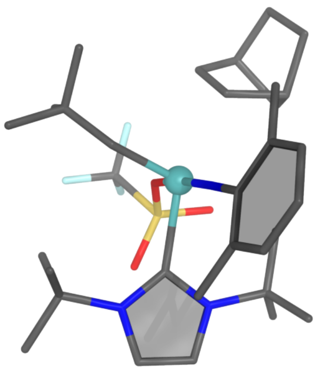

Figure S54. BP86/def2-SVP/COSMO (ε = 9.0) optimized cationic **adduct VI** structure of **2**.

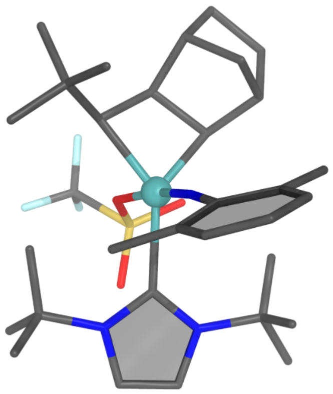

Figure S55. BP86/def2-SVP/COSMO ($\varepsilon = 9.0$) optimized **metallacyclobutane I** structure of **2.**

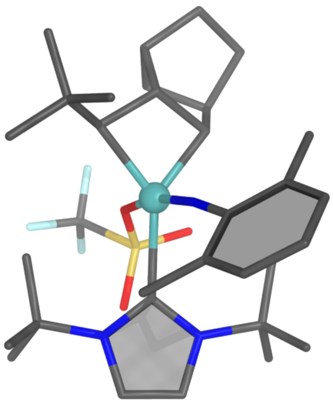

Figure S56. BP86/def2-SVP/COSMO ($\varepsilon = 9.0$) optimized **metallacyclobutane II** structure of **2.**

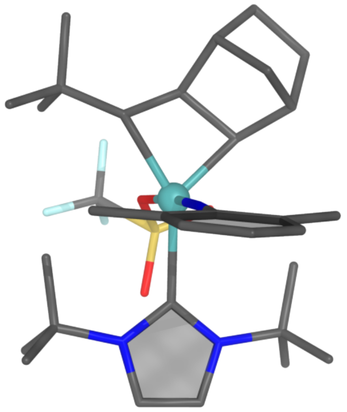

Figure S57. BP86/def2-SVP/COSMO ($\varepsilon = 9.0$) optimized **metallacyclobutane III** structure of **2.**

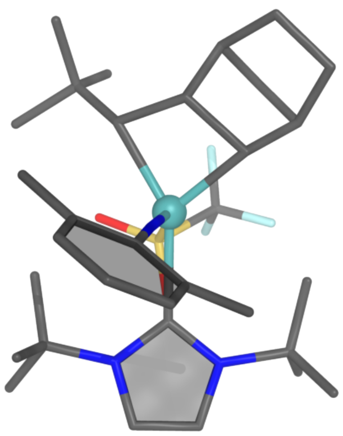

Figure S58. BP86/def2-SVP/COSMO (ε = 9.0) optimized **metallacyclobutane VI** structure of **2.**

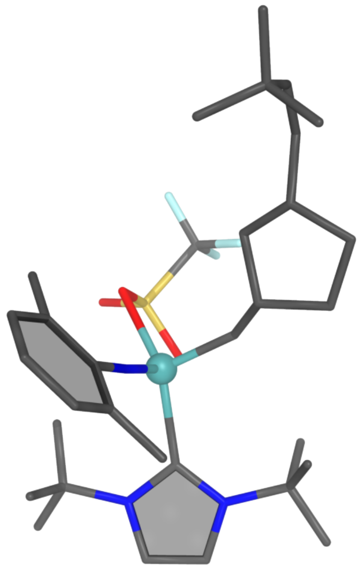

Figure S59. BP86/def2-SVP/COSMO (ε = 9.0) optimized structure of the *syn*+1_{cis} conformer (**product I**) of **2**.

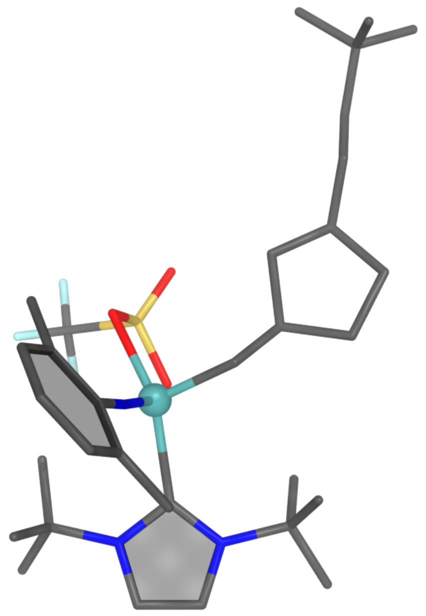

Figure S60. BP86/def2-SVP/COSMO ($\varepsilon = 9.0$) optimized structure of the *anti*+1_{trans} conformer of **2** (**product II**). The most stable conformation was identified after extensive conformer search (see Computational Methodology for details).

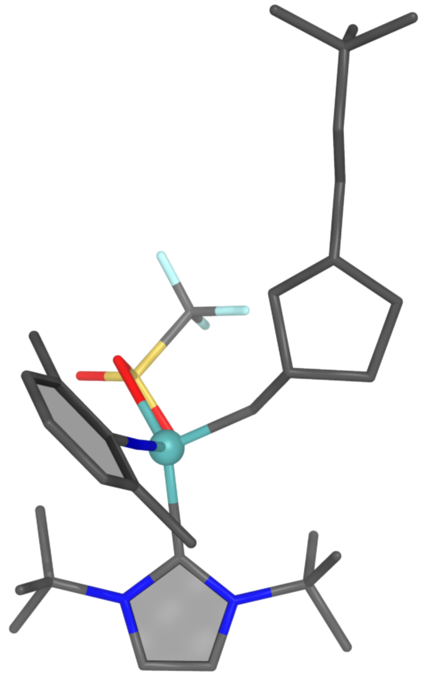

Figure S61 BP86/def2-SVP/COSMO ($\varepsilon = 9.0$) optimized structure of the *syn*+1_{trans} conformer of **2** (**product III**).

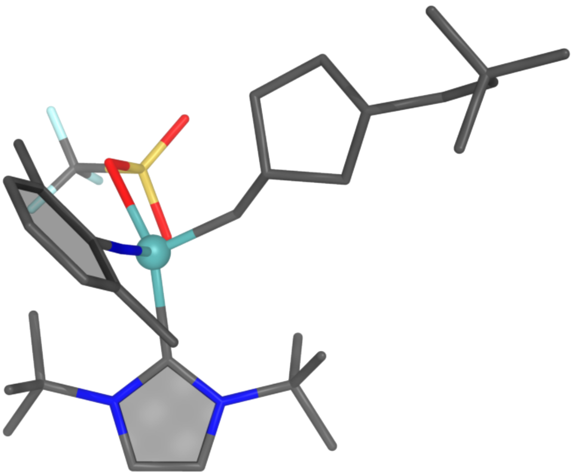

Figure S62. BP86/def2-SVP/COSMO (ε = 9.0) optimized structure of the *anti*+1_{cis} conformer of **2** (**product IV**). The most stable conformation was identified after extensive conformer search (see Computational Methodology for details).

References

1. Johnson, E. R.; Keinan, S.; Mori-Sanchez, P.; Contreras-Garcia, J.; Cohen, A. J.; Yang, W. T., Revealing Noncovalent Interactions. *J. Am. Chem. Soc.* **2010,** *132* (18), 6498-6506.
2. Contreras-Garcia, J.; Johnson, E. R.; Keinan, S.; Chaudret, R.; Piquemal, J. P.; Beratan, D. N.; Yang, W. T., NCIPLOT: A Program for Plotting Noncovalent Interaction Regions. *J. Chem. Theory Comput.* **2011,** *7* (3), 625-632.
3. Humphrey, W.; Dalke, A.; Schulten, K., Vmd: Visual Molecular Dynamics. *J. Mol. Graph. Model.* **1996,** *14* (1), 33-38.
4. Herz, K.; Podewitz, M.; Stöhr, L.; Wang, D. R.; Frey, W.; Liedl, K. R.; Sen, S.; Buchmeiser, M. R., Mechanism of Olefin Metathesis with Neutral and Cationic Molybdenum Imido Alkylidene N-Heterocyclic Carbene Complexes. *J. Am. Chem. Soc.* **2019,** *141* (20), 8264-8276.
5. Buchmeiser, M. R.; Sen, S.; Unold, J.; Frey, W., N-Heterocyclic Carbene, High Oxidation State Molybdenum Alkylidene Complexes: Functional-Group-Tolerant Cationic Metathesis Catalysts. *Angew. Chem. Int. Ed.* **2014,** *53* (35), 9384-9388.
